# Genome-Wide Identification of Tannase Genes and Their Function of Wound Response and Astringent Substances Accumulation in Juglandaceae

**DOI:** 10.3389/fpls.2021.664470

**Published:** 2021-05-17

**Authors:** Jianhua Wang, Ketao Wang, Shiheng Lyu, Jianqin Huang, Chunying Huang, Yulin Xing, Yige Wang, Yifan Xu, Peipei Li, Junyan Hong, Jianwei Xi, Xiaolin Si, Hongyu Ye, Yan Li

**Affiliations:** State Key Laboratory of Subtropical Silviculture, Zhejiang A&F University, Hangzhou, China

**Keywords:** tannase, Juglandaceae, hydrolyzable tannins, astringency, phylogenetic analysis, expression profile, leaf injury

## Abstract

Tannins are important polyphenol compounds with different component proportions in different plant species. The plants in the Juglandaceae are rich in tannins, including condensed tannins and hydrolyzable tannins. In this study, we identified seven tannase genes (*TAs*) responsible for the tannin metabolism from walnut, pecan, and Chinese hickory, and three nut tree species in the Juglandaceae, which were divided into two groups. The phylogenetic and sequence analysis showed that *TA* genes and neighboring clade genes (*TA-like* genes) had similar sequences compared with other carboxylesterase genes, which may be the origin of *TA* genes produced by tandem repeat. *TA* genes also indicated higher expressions in leaf than other tissues and were quickly up-regulated at 3 h after leaf injury. During the development of the seed coat, the expression of the synthesis-related gene *GGTs* and the hydrolase gene *TAs* was continuously decreased, resulting in the decrease of tannin content in the dry sample of the seed coat of Chinese hickory. However, due to the reduction in water content during the ripening process, the tannin content in fresh sample increased, so the astringent taste was obvious at the mature stage. In addition, the *CcGGTs*’ expression was higher than *CiGGTs* in the initiation of development, but *CcTAs* continued to be down-regulated while *CiTA2a* and *CiTA2b* were up-regulated, which may bring about the significant differences in tannin content and astringent taste between Chinese hickory and pecan. These results suggested the crucial role of *TAs* in wound stress of leaves and astringent ingredient accumulation in seed coats of two nut tree species in the Juglandaceae.

## Introduction

Tannins are the fourth largest plant secondary metabolites after cellulose, hemicellulose, and lignin (Lekha and Lonsane, 1997). They are plant polyphenols with a large molecular weight widely distributed in various plant organs (Salminen, 2018). Classically, tannins are mainly divided into condensed and hydrolyzable tannins. Condensed tannins are polymers of flavan-3-ol and/or flavan-3,4-diol, also known as proanthocyanidins because their acidic hydrolysates are anthocyanidins (de Jesus et al., 2012; Combs, 2016); hydrolyzable tannins are gallate esters of polyols (usually D-glucose). Hydrolyzable tannins can be further divided into gallotannins and ellagitannins, and the latter will produce ellagic acid after being hydrolyzed while the former will not (Lamy et al., 2016). It is the most important characteristic of tannins that there are a large number of hydroxyl groups, which can bind to proteins in the form of hydrogen bonds, causing changes in protein conformation. Tannins also play an important role in biotic and abiotic stresses of plants, such as protecting plants from predators and pathogens (Treutter, 2006; Nakabayashi et al., 2014). In addition, a variety of phenolic substances in tannins also have great impacts on human health.

Tannase (TA), also known as tannin acyl hydrolase (EC 3.1.1.20), could hydrolyze galloyl ester bonds in hydrolyzable tannins and other gallate esters but does not act on condensed tannins (Zhang et al., 2019). Tannases were mainly found in microorganisms such as fungi and bacteria, and the enzymatic properties and protein structure of microbial tannases have been extensively studied (Ren et al., 2013; Jana et al., 2014; Abdel-Naby et al., 2016). At present, there are few studies on plant tannases: some researchers found the phenomenon of substrate hydrolyzed via the enzymatic tests *in vitro* on UDP-glucose-dependent glycosyltransferases related to gallate tannin biosynthesis, which may be due to some unknown esterase involvement (Weisemann et al., 1988; Cammann et al., 1989; Denzel and Gross, 1991). Subsequently, Niehaus and Gross (1997) isolated and purified this esterase from leaves of pedunculate oak (*Quercus robur*), which indeed could hydrolyze galloylglucose and was similar to fungal tannase, and classified it as plant tannase. However, the gene encoding plant tannase has not been characterized for many years due to the limitation of analytical techniques. Recently, plant *TA* genes from tea trees were first discovered and characterized, and *TA* genes were also distributed in some plants rich in tannins (Dai et al., 2020).

The tree species of Juglandaceae such as walnut (*Juglans regia*), pecan (*Carya illinoinensis*), and Chinese hickory (*Carya cathayensis*), as well as some precious timber species such as black walnut (*Juglans nigra*) have high economic values (Huang et al., 2019). They have been cultivated, domesticated, and utilized by human beings for a long time (Guo et al., 2020). The plants in the Juglandaceae are rich in tannins, both hydrolyzable and condensed tannins, especially in seeds, barks, and roots (Gong and Pegg, 2017; Jia et al., 2018; Jahanban-Esfahlan et al., 2019). However, there are some differences in the composition of tannins among different species in seeds: walnut and Chinese hickory nuts may have more hydrolyzable tannins, especially ellagitannins, while pecan has more condensed tannins (Regueiro et al., 2014; Gong and Pegg, 2017).

It is known that there are *TA* genes in walnut, which may regulate tannin composition (Dai et al., 2020), and whether other species in the Juglandaceae have *TA* genes has not been reported. The study of *TA* genes in the Juglandaceae can help us further understand the important role of tannins in the difference in astringent taste between different species in the Juglandaceae. In this study, we systematically identified *TA* genes and other homologous genes in nine plants based on the published genomic and transcriptional data. Seven *TA* genes were identified from walnut, pecan, and Chinese hickory, three important nut tree species in the Juglandaceae. Their motif composition, gene structure, chromosome localization, and miRNA prediction were comprehensively analyzed. At the same time, we measured the expression levels of *TA* genes in roots, stems, leaves, flowers, peels, testae (seed coats), and embryos. The expression changes of GGTs and TAs in response to leaf injury stress were further analyzed. Finally, the combination of RT-qPCR and HPLC results led us to discover the relationship between *TA* genes and tannin content changes during fruit development, preliminarily explaining the formation of the difference in astringency between Chinese hickory and pecan. These results revealed that *TA* genes may play a crucial role in the metabolism of tannins in the Juglandaceae, which will be good for future tannin research of other plants.

## Materials and Methods

### Plant Materials and Growth Conditions

Chinese hickory (landrace “ZAFU-1”) and pecan (cultivar “Mahan”) plants, planted at the farm of Zhejiang Agriculture and Forestry University (Hangzhou, China), were used as experimental materials. Mature female flowers were collected from late April to early May, and roots, stems, buds, leaves, peels, embryos, and testae (seed coats) were collected in June to October. In order to mimic the effect of chemical defenses on herbivory, the upper third compound leaves of each leaflet were cut off with scissors before and after treatment for 3, 6, 12, 24, and 48 h, respectively. For the tannin determination of seed coat, we collected five periods of Chinese hickory (CcS1–CcS5) as well as ripe period pecan and walnut (CiS5 and JrS5). The walnut (cultivar “Xiangling”) was sampled from Tiantongyuan Company (Tianshui, Gansu, China). All plant samples were frozen in liquid nitrogen prior to storage at −80°C until use.

### Identification of *TA* Genes in Juglandaceae and Other Plants

The genomes of Chinese hickory (*C. cathayensis, Cc*) and pecan (*C. illinoinensis, Ci*) were downloaded from the GIGADB database^1^, and the genome of walnut (*J. regia, Jr*) was downloaded from Xuehui Huang Lab^2^. The CsTA protein sequence of tea (*Camellia sinensis*, *Cs*) was downloaded from the National Center for Biotechnology Information (NCBI). The genome and protein sequence of other species was downloaded from the Phytozome database, including clementine (*Citrus clementina, Ccl*), persimmon (*Diospyros kaki, Dk*), strawberry (*Fragaria* × *ananassa, Fa*), woodland strawberry (*F. ananassa, Fv*), pomegranate (*Punica granatum, Pg*), aspen (*Populus tremula, Pt*), and grape (*Vitis vinifera, Vv*). The protein of CsTA was used as a query to search against the protein database of other plants, and the putative proteins were obtained by BLASTP search with a bit score of more than 200 and an *E*-value less than 1 × 10^–20^. For Chinese hickory and pecan, in order to obtain all potential *TA* genes, the protein sequence of CsTA was also used to blast the novel transcripts from previous transcriptome data. All potential TA protein sequences were examined by searching for abhydrolase_3 (PF07859) and COesterase (PF00135) domains using the Pfam database^3^ and SMART database^4^ (Letunic et al., 2012; Finn et al., 2016). All candidate sequences were searched on the whole genome to find the possible genome location of *TA* genes through TBLASTN.

### Analysis of Protein Sequence Properties

The characteristic of TA proteins, including molecular weight (MW), isoelectric points (pI), and grand average of hydropathicity (GRAVY) were predicted by ExPASy ProtParam^5^ (Gasteiger, 2003). Additionally, we, respectively, predicted the signal peptides and transmembrane (TM) domains with SignalP 4.0^6^ and TMHMM 2.0^7^ (Krogh et al., 2001; Petersen et al., 2011).

### Sequences Alignment and Phylogenetic Analysis

The protein sequences of previously reported tannase (from plant, bacterial, and fungal) and homologous genes were obtained from the NCBI protein database (Dai et al., 2020). The potential TA protein sequences in the other published genomes were identified by the method described earlier. The multiple sequence alignment of all proteins was performed using MAFFT version 7^8^ (Nakamura et al., 2018). A maximum likelihood (ML) phylogenetic tree of full-length protein of all sequences was constructed with 1,000 bootstrap replicates using MEGA10.0 (Tamura et al., 2011). The best model “WAG + G” was predicted by MEGA10.0 due to its lowest Bayesian Information Criterion (BIC) score.

### Analysis of Gene Structure, Conserved Motif, and *Cis*-Acting Elements

The conserved motifs of TAs were searched using MEME 5.1.1^9^ (Bailey et al., 2009). It was performed with the following parameters: 20 different motifs, a motif width of 6–50 amino acids, and any number of repetitions. The potential function of motifs was searched against Pfam database (see Text Footnote 3). Plant CARE software^10^ was used to predict the *cis*-acting elements within 2000 bp upstream of all TA genes (Lescot, 2002), and PLACE^11^ was used as a supplement to *cis*-acting elements of brassinosteroid and cytokinin (Higo et al., 1999). The illustrations of gene structures, motifs, and *cis*-acting elements were then generated using TBtools (Chen et al., 2020).

### MiRNA Predicted in the Juglandaceae *TA* Genes

The psRNATarget Server^12^ was used to search and predict potential miRNAs of the coding sequences of the Juglandaceae *TA* genes with default parameters (Dai and Zhao, 2011). Cytoscape software was used to visualize the predicted interaction between miRNA and TA genes in Juglandaceae (Shannon, 2003).

### Expression Analysis of *TA* Genes

Total RNA was extracted from the samples using the Quick RNA isolation Kit (Huayueyang, China), and cDNA was synthesized using the PrimeScript1st^TM^ Strand cDNA Synthesis Kit (Takara, Japan) according to the manufacturer’s instructions. The expression levels of *TA* genes in the roots, stems, leaves, female flowers, peel, young embryos, mature embryos, and seed coats at five developmental stages were measured by RT-qPCR using the CFX96 real-time PCR Detection system (Bio-Rad, United States) with TB Green^®^ Premix Ex Taq (TaKaRa, Japan). The reaction condition was 95°C for 3 min, followed by 40 cycles at 95°C for 10 s, and 55°C for 30 s. Meanwhile, the expression levels of *TA* genes treated with mimicking herbivory were also measured with the same method. The RT-qPCR primers of *TA* genes listed in Supplementary Table 5 were obtained by online software primer 3^13^. The relative expression was calculated based on the 2^–Δ^ ^CT^ method (Livak and Schmittgen, 2001), and the expression of actin gene, which had the same sequences in Chinese hickory and pecan, was monitored as an internal control. Three biological replicates were performed in the RT-qPCR experiment, and three technical replicates were performed in each biological replicate.

### SDS-PAGE Analysis of Salivary Protein Precipitated by Seed Coats Extracts

Saliva was collected from six healthy non-smoking volunteers and 2 ml of saliva from each volunteer was used to make a saliva pool (whole saliva). The collection time was standardized from 2 to 3 pm to reduce the concentration changes associated with circadian rhythm secretion. Samples were collected by draining saliva into a cold tube. All samples were collected and centrifuged at 4,000 *g* for 20 min at 4°C to remove all insoluble matter. The obtained supernatant was divided equally and immediately frozen at −80°C, which was called whole saliva (WS) (Ramos-Pineda et al., 2020).

The 20 mg of seed coat lyophilized sample was dissolved in 1 ml of distilled water as the sample solution, and the concentration of which was noted as 20 mg seed coat dry weight/ml and diluted with distilled water to three concentrations (0.625, 1.25, and 2.5 mg/ml). The 200 μl of whole saliva was mixed with 200 μl of seed coat extracts or distilled water, followed by vortexing for 10 s, and incubating for 20 min at 37°C. The mixtures were centrifuged at 12,000 *g* for 5 min, and the precipitate was discarded. Then one-quarter volume of 5× sample loading buffer (250 mM Tris-HCl, 10% SDS, 0.5% Bromophenol blue, and 50% Glycerol, pH 6.8) was added to the supernatant.

The detection of salivary proteins after reaction with seed coat extracts was performed by SDS-PAGE using the DYCZ-24B vertical electrophoresis system (LiuYi, China). Forty microliters of each treated sample was electrophoresed on an 8 cm × 7 cm and 1.5-mm-thick, 13.5% w/v denaturing polyacrylamide gel, covered with a 5% w/v polyacrylamide stacking gel. Protein markers (Bio-Rad, United States) in the molecular weight range (10–250 kDa) were also loaded. Electrophoresis is performed at a constant voltage of 75 V for 30 min, followed by switching the voltage to 120 V until the tracking dye (bromophenol blue) reaches the bottom of the gel. The gels were stained with Coomassie Brilliant Blue R250 Staining Solution (0.2% Coomassie blue R250, 10% acetic acid, and 45% methanol) and rinsed overnight using a destaining solution (10% acetic acid and 23.75% ethanol).

### Astringency Evaluating Assay

An astringency evaluation method based on the precipitation of tannins by protein was used to evaluate astringency, modified from Llaudy et al. (2004) and Jauregi et al. (2016). We modified this method by replacing ovalbumin with bovine serum albumin (BSA) and adjusting the buffer. Solutions of BSA at 0.4–3.2 mg/ml, solutions of tannic acid at 0.2–1.0 mg/ml, and 2.0 mg/ml seed coat extracts were prepared using 100 mM acetate buffer solution (pH = 5.0), respectively. Two hundred microliters of tannic acid solution and 200 μl of ovalbumin solution were mixed and vortexed for 10 s, and after 10 min, 12,000 *g* was separated for 10 min. One hundred microliters of supernatant was diluted 50-fold, and the absorbance value at 280 nm was detected using a UV-2600 UV-vis spectrophotometer (Shimadzu, Japan).

### Determination of Seed Coat Phenolic Compounds

After manual peeling, the seed coats were freeze-dried for 48 h with a lyophilizer (Christ Alpha 2–4 LD plus, Germany) and ground into powder. Take 20 mg of the sample, add 1.4 ml of 80% aqueous acetone solution, and leave it overnight at 4°C, followed by ice bath of ultrasonic extraction for 2 h. After centrifugation (12,000 *g*, 5 min), the supernatant was concentrated in a Rotational Vacuum Concentrator (RVC 2-25 CD plus, Germany) for 1 h to remove acetone, the sample residue was extracted once with a new aqueous acetone solution, and the supernatant was pooled twice and vacuum concentrated to remove acetone. The sample solution was then freeze-dried for 24 h to obtain the lyophilized powder, and 1 ml of methanol was added to redissolve it.

We performed a simple analysis of the samples for hydrolyzable and condensed tannins according to the method described by Gong and Pegg (2017). For the analysis of hydrolyzable tannins, a 1,260 series HPLC system (Agilent Technologies, Inc., Wilmington, DE, United States) was used for developing the chromatographic conditions with a 150 mm × 4.6 mm i.d., 2.6 μm, Kinetex PFP column with a pore size of 100 Å (Phenomenex, Torrance, CA, United States). Ten microliters of sample solution were injected after being filtered through a 0.45-μm PTFE membrane. Mobile phases consisted of H_2_O/CH_3_CN/CH_3_COOH (94:5:1, v/v/v) (solvent A) and H_2_O/CH_3_CN/CH_3_COOH (59:40:1, v/v/v) (solvent B). A linear gradient elution at a flow rate of 0.8 ml/min was run as follows: 0–30 min, 0–60% B; 30–32 min, 60% B; 32–33 min, 60-100% B; 33–35 min, 100–0% B. Detection wavelengths were 255 nm (i.e., ellagic acid and its derivatives) and 280 nm (phenolic acids, catechin, and epicatechin). Tentative identification of separated components was achieved by matching UV/vis spectra and retention times (*t*_R_) with standard compounds.

Condensed tannins were separated using the same Agilent chromatograph but with a 150 mm × 4.6 mm i.d., 3 μm, Luna HILIC column with a pore size of 200 Å (Phenomenex). Similarly, 10 μl of filtered sample solution was injected. Mobile phases consisted of CH_3_CN/CH_3_COOH (98:2, v/v) (solvent A) and CH_3_COOH/H_2_O/CH_3_CN 95:3:2, v/v/v) (solvent B). A linear gradient elution at a flow rate of 1 ml/min was run as follows: 0–25 min, 0–45% B; 25–30 min, 45–0% B. The excitation/emission wavelengths for fluorescence detection were set at 276/316 nm, respectively. Procyanidin A2, B1, B2, and C1 were employed to map the *t*_R_ values.

### Subcellular Localization Analysis

The protein subcellular localization was performed by *Agrobacterium tumefaciens*-mediated transient expression in *Nicotiana benthamiana* leaves. The full-length CDS of TAs was amplified using the gene-specific primers and subcloned into pENTR-D-TOPO (Invitrogen, United States). Sequences of *CiTA2a* and *CiTA2b* were highly similar, and one universal primer was designed for analysis. After validation by the sequencing, full-length TAs were cloned into pK7FWG2 vector with EGFP reporter gene by LR reaction. The recombinant plasmids were introduced into *A. tumefaciens* strains GV3101 competent cell and cultured on the LB medium with 50 μg/ml gentamicin (Geta), 50 μg/ml rifampicin (Rif), and 50 μg/ml kanamycins (Kana) at 28°C in the constant temperature incubator. Two days later, a single colony was transferred into lipid LB medium and cultured for another 2 days at 28°C. Then, the cultures of *A. tumefaciens* (OD600 = 0.5–0.6) were centrifuged at 5,000 rpm at room temperature for 10 min and re-suspended in MMA buffer (10 mM MES, 10 mM MgCl_2_, and 200 μM acetosyringone, pH = 5.6) to an OD_600_ of 1.0 and then incubated at room temperature in the darkness for 2–3 h. Subsequently, we injected the agrobacterium cultures into the 4-week-old and well-growing *N. benthamiana* leaves using a 1-ml syringe. Two days after the culture, GFP fluorescence was observed and examined using laser confocal fluorescence microscopy (excitation: 488 nm; emission: 495–515 nm) (LSM 800, Zeiss, Germany). The experiments were repeated three times.

### Statistical Analysis

For the expression of each gene in the figures, multiple comparisons among different samples were performed using Tukey’s honestly significant difference (Tukey’s HSD) with HSD.test function in R package “agricolae.” Different letters above the columns indicate statistically significant differences between groups (*P* < 0.05).

## Results

### Identification and Characterization of *TA* Genes in the Juglandaceae

**FIGURE 1:**
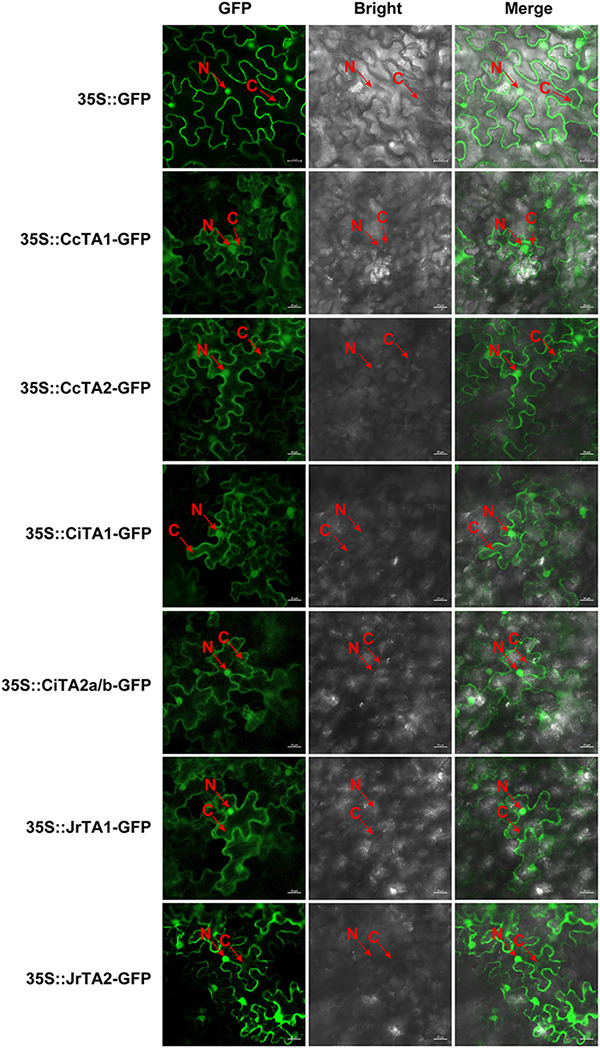
Subcellular localization analysis of *TA* genes in Juglandaceae. The protein subcellular localization was performed by *Agrobacterium tumefaciens*-mediated transient expression in *N. benthamiana* leaves. GFP fluorescence was observed and examined using a laser confocal fluorescence microscopy (excitation: 488 nm; emission: 495–515 nm) (LSM 800, Zeiss, Germany). Scale bar in images is 20 μm. Red arrows indicate important areas (N stands for the nucleus and C stands for the cytoplasm).

Protein blast results revealed that many proteins showed high identification with CsTA in each plant species. Among the similar sequences, we excavated 7 *TA* genes from the genome of Chinese hickory, pecan, and walnut (Table 1 and Supplementary Tables 1, 2). The results indicated that walnut and Chinese hickory had two *TA* genes, while pecan had three *TA* genes. These TA proteins in length ranged from 303 to 368 amino acids, with molecular weights from 33.21 to 40.49 kDa and theoretical isoelectric points ranging from 5.52 to 6.17. The average protein length, MW, and hydrophilicity in pomegranate are bigger than in other species. The average pI value of 6.055 in walnut is relatively bigger than others, but the average pI is only 5.51 in strawberry. The GRAVY value of all TA proteins was shown to be less than 0 (varying from −0.307 to −0.111), indicating their hydrophilic feature. Compared with other species, pecan and pomegranate both had a protein, *CiTA1* and *PgTA2*, which had a significantly longer length, bigger molecular weights, and lower PI. Subcellular localization analysis indicated that the vast majority of *TA* genes are localized in the cytoplasm, except *CiTA1* and *PgTA2* are located in the plastid (Table 1). The results of signal peptide analysis indicated that only *CiTA1* and *PgTA2* contained an N-terminal signal peptide, and TM domain analysis showed that all of TAs do not possess TM domains. To determine the subcellular localization of TA genes, the TA-EGFP fusion proteins in the tobacco leaves were examined under a confocal microscope. As shown in Figure 1, the TA-GFP fluorescent signals of all tannase proteins in three species were observed in the cytosol and nucleus. This result indicated that TAs may be predominantly hydrolyzed substrates in the cytosol and nucleus.

**TABLE 1:** Detailed information of plant tannase genes in the Juglandaceae and other land plants.

**Gene**	**Gene ID**	**Length (aa)**	**pI**	**MW (kDa)**	**GRAVY**	**Localization**
*CcTA1*	BGI_novel_G001926	303	5.99	33.21	−0.141	Cytoplasm
*CcTA2*	BGI_novel_G001927	306	5.65	33.53	−0.245	Cytoplasm
*CiTA1*	CIL1404S0013	368	5.89	40.49	−0.113	Plastid
*CiTA2a*	BGI_novel_G000346	306	5.72	33.55	−0.231	Cytoplasm
*CiTA2b*	BGI_novel_G000348	306	5.52	33.55	−0.235	Cytoplasm
*JrTA1*	JreChr05G12349	303	6.17	33.53	−0.307	Cytoplasm
*JrTA2*	JreChr05G12348	306	5.94	33.59	−0.123	Cytoplasm
*CsTA1*	CSS0013720	299	5.61	33.03	−0.187	Cytoplasm
*CsTA2*	CSS0029502	299	5.41	33.07	−0.182	Cytoplasm
*CsTA3*	CSS0031888	299	5.62	33.13	−0.252	Cytoplasm
*CsTA4*	CSS0037283	299	5.61	33.14	−0.261	Cytoplasm
*CsTA*	MK381269	299	5.61	33.09	−0.256	Cytoplasm
*CclTA*	MK381273	305	5.56	33.56	−0.266	Cytoplasm
*DKTA*	Dlo_pri0202F.1_g00930	303	5.81	32.90	−0.111	Cytoplasm
*FaTA*	MK381272	306	5.50	33.50	−0.135	Cytoplasm
*FvTA*	FvH4_2g06620	306	5.51	33.46	−0.134	Cytoplasm
*PgTA1*	LOC116202495	310	5.56	34.68	−0.304	Cytoplasm
*PgTA2*	LOC116204819	358	5.86	39.45	−0.275	Plastid
*PtTA1*	Potri.009G105000	303	5.99	33.01	−0.196	Cytoplasm
*PtTA2*	Potri.004G143200	301	5.25	33.00	−0.143	Cytoplasm
*VvTA*	VIT_03s0063g00830	302	5.59	33.15	−0.129	Cytoplasm

**Cc, Carya cathayensis; Ci, Carya illinoinensis; Jr, Juglans regia; Cs, Camellia sinensis; Ccl, Citrus clementina; DK, Diospyros kaki; Fa, Fragaria ananassa; Fv, Fragaria ananassa; Pg, Punica granatum; Pt, Populus tremula; Vv, Vitis vinifera*.*

### Sequence Alignment and Phylogenetic Analysis

**FIGURE 2:**
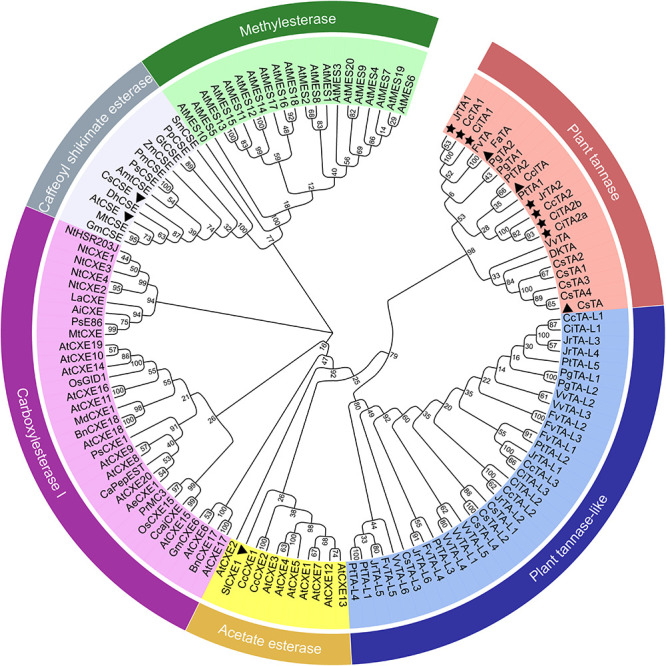
Molecular phylogenetic analysis of gene families among plant tannase genes and other carboxylesterase genes. The evolutionary history was inferred using the Neighbor-Joining method. The bootstrap consensus tree inferred from 1000 replicates was used to represent the evolutionary history of the tannase genes. Whole tree was divided into five clades, plant tannase clade (with red range), plant tannase-like clade (with blue range), acetate esterase clade (with yellow range), carboxylesterse I clade (with orchid range), caffeoyl shikimate esterase clade (with gray range), and methylesterase clade (with green clade). Previous reported tannase and carboxylesterase genes were marked with a triangle. The Juglandaceae tannase genes in walnut, pecan, and Chinese hickory were marked with asterisks.

To examine the phylogenetic relationships of plant tannases, a phylogenetic tree of plant carboxylases to which the tannases belonged was constructed (Figure 2). In the present study, plant carboxylesterases were divided into five clades: methyl esterase, caffeoyl shikimate esterase, carboxylesterase I, acetate esterase, plant tannase, and plant tannase-like. The entire phylogenetic tree was divided into three major clades, with caffeoyl shikimate esterase and methylesterase as the first clade, carboxylesterase I as the second clade, and acetate esterase, plant tannase, and plant tannase-like as the third clade. According to protein blast results, except tannases, all genes that shared high identify with *CsTA* belonged to plant tannase-like clade.

The methyl esterase evolutionary branch contains AtMES1-20 from *Arabidopsis thaliana* (Yang et al., 2008). The methyl esterase clade was thought to be related to the hydrolyzable MeJA, MeSA, and MeIAA. Caffeoyl shikimate esterase contains AtCSE, CsCSE, and others involved in lignin formation (Vanholme et al., 2013; Dai et al., 2020). Other carboxylesterase genes could be split into four clades. The typical carboxylesterase grouped carboxylesterase I clade contained numerous genes. Phylogenetic analysis showed that plant tannase, plant tannase-like, and acetate esterase clade were closest to the carboxylesterase I clade. These results suggested that these three clade genes may be derived from carboxylesterase I genes and tannase and tannase-like genes may be derived from acetate esterase genes.

In plant tannase clade, most species contained more than one tannase gene and we found 5 *TA* genes in tea. *CsTA* was reported in a previous study and *CsTA1–4* had not been reported. *CsTAs* were grouped with *TAs* in grape and persimmon, which was consistent with their species evolutionary status. In the other group, *TAs* are further divided into two classes. *TAs* in strawberry, clementine, and aspen all belong to class 1, while *TAs* in pomegranate all belong to class 2. Interestingly, for 3 Juglandaceae species (walnut, Chinese hickory, and pecan), all have different *TA* genes assigned to two classes. This classification may lead to differences in tannase functions in Juglandaceae, such as different catalytic efficiencies for different substrates, or produce different metabolites.

### Structure and Conserved Motif Analysis of *TA* Genes

**FIGURE 3:**
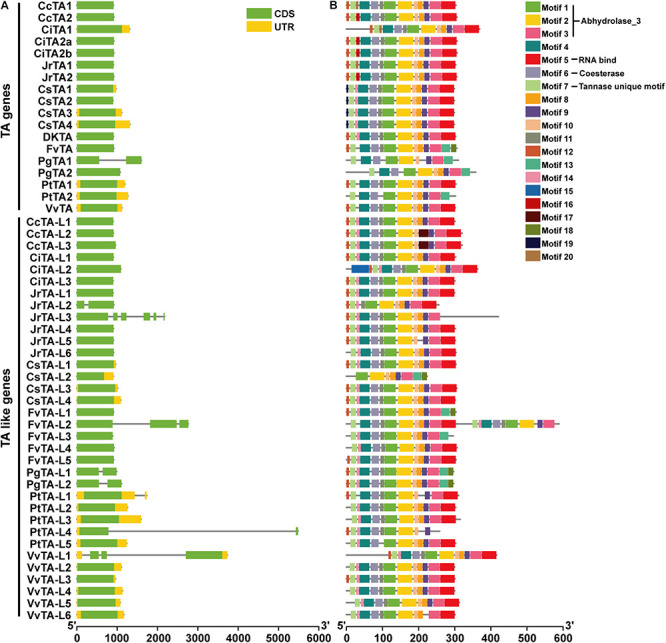
Structure analysis of *TA* and *TA*-like genes in plants. **(A)** Exon–intron structures of *TA* and *TA*-like genes in 13 species. CDSs were shown as green boxes, introns were shown as thin gray lines, and UTRs are shown as yellow boxes. **(B)** Distribution of conserved motifs among proteins identified using MEME suite program. The motifs, numbered 1–20, were designated with a specific color. The sequence information for each motif was provided in Supplementary Table 3.

The exon–intron structure of *TA* genes was analyzed based on the cDNA and DNA sequences. Results showed that almost all *TA* genes only contained one exon in different species (Figure 3). The similar single-exon structure was also observed in *TA-like* genes, and about 91.11% of them are intronless. Only nine genes have more than one exon including one *TA* gene in pomegranate and eight *TA-like* genes. In walnut, *JrTA-L2* and *JrTA-L3* had two and six exons, respectively. In aspen and pomegranate, two genes contained two exons. In strawberry and grape, each had one gene that contained more than one exon.

To understand the diversity of motif compositions among different tannase proteins, the conserved motifs were predicted using MEME. Motifs 1, 2, 3, 4, 6, 7, 8, 9, and 10 were almost distributed in each TA and TA-like protein. Among these nine motifs identified, Motifs 1, 2, and 3 corresponded with abhydrolase_3 domain (PF07859) and Motif 6 corresponded with carboxylesterase domain (PF00135). Motif 5 was also a broadly distributed motif that may play a role in RNA binding and not found in the genes in strawberry, pomegranate, and aspen. According to a previous study (Dai et al., 2020), Motif 7 corresponded with the tannase conserved motif. In alignment result, we also found similar motif 7 in TA-like family, but existed 2 major amino acids change which may lead to their function differentiation (Supplementary Figure 1). Comparing the TA proteins with the TA-like proteins, we found that Motif 14 was more common in TA-like proteins (97.06%) than in TA proteins (33.33%) and this motif was found in all TA proteins of tea and persimmon (Supplementary Table 3). However, Motifs 16, 19, and 20 are found in TA proteins (22.22, 22.22, and 16.67%), but not in any TA-like protein. Motifs 16 and 20 were species unique motifs of TA proteins in Juglandaceae species (walnut, pecan, and Chinese hickory). Motif 16 was only found in TAs of class 2 in Juglandaceae and Motif 20 was in class 1. Motifs 15 and 17 were *Carya* genus in Juglandaceae. Motif 19 existed in all TA proteins in tea. Further analysis of the three motifs revealed that genes containing Motif 19 are all in tea. These motifs have not found corresponding known domains, which may lead to differences in tannase function in different species.

### Analysis of the Promoter *Cis*-Acting Regulatory Elements of *TA* Genes

**FIGURE 4:**
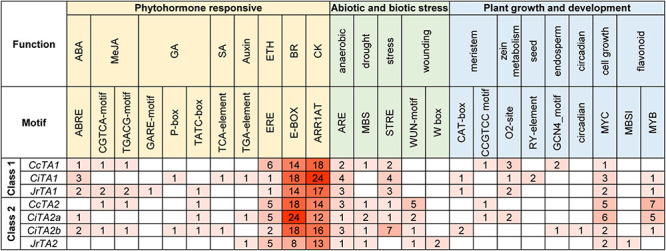
*Cis*-acting element analysis of *TA* gene promoter regions in Juglandaceae.

The variances of tannase motif in Juglandaceae may result in the different enzyme activity. The distribution of different *cis*-acting elements in gene promoters may indicate the differences in their function and regulation when environmental stresses are encountered. To understand the regulatory element of tannases in Juglandaceae, we examined all *TA* and *TA*-like genes promoter *cis*-elements, 2-kb upstream of the ATG start in walnut, pecan, and Chinese hickory (Figure 4 and Supplementary Figure 2). All regulatory elements were grouped into three categories by function, phytohormone responsive, abiotic and biotic stress, and plant growth and development.

The number of motifs between three genes of class 1 and 4 genes of class 2 is almost the same. *JrTA2*, which was the least motif, only had 35 predicted regulatory elements. The most common motifs found in promoter were E-box (involved in the brassinolide responsiveness) and ARR1AT (involved in the cytokinin responsiveness). Two motifs, WUN-motif and W-box, are involved in wound-responsive element belonging to abiotic stress and were only found in the gene of class 2. Flavonoid biosynthetic-related motif (MYB) and cell growth promotion-related motif (MYC) were significantly higher in two genes, *CcTA2* and *CiTA2a*. *CiTA2b* has more stress-responsive element (STRE) than other genes. This result demonstrates that some identified *cis*-elements in tannase genes may be involved in phytohormone regulation, wounding, and so on. Two classes of tannase genes may have different regulation ways.

### Prediction Target Network of *TA* Genes and MicroRNA in Chinese Hickory

**FIGURE 5:**
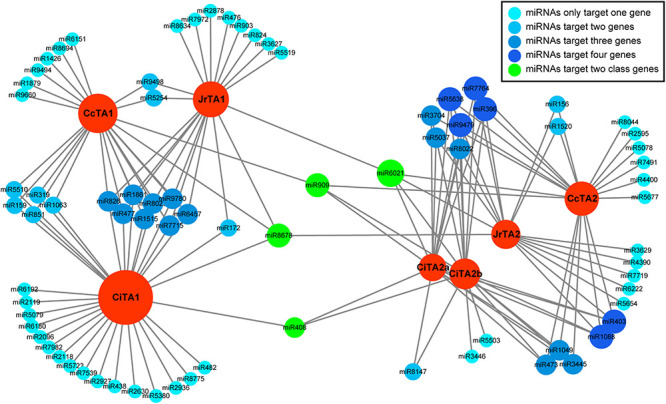
Target network between *TAs* and potential miRNAs in Juglandaceae. Red circles represented *TA* genes; other circles denoted potential miRNAs, and different colors indicated the co-regulation ability.

MicroRNA is a very important mechanism for post-transcriptionally regulation. In order to find the candidate miRNA of *TA* genes, we predicted the target relationship with psRNAtarget using all plant miRNAs (Supplementary Table 4). The result showed that each *TA* gene contained multiple sequences that could well-match with miRNA and might be the targets of miRNAs (Figure 5). In total, there were 78 miRNAs that were predicted as candidate regulators of *TA* genes in walnut, pecan, and Chinese hickory. The average number of predicted miRNA in each gene was 21 and *CiTA1* had the most miRNA target sites. From the result, we found that most miRNAs were found in different *TA* genes and only a small percentage of miRNAs was unique to each gene. The targeted network showed that two classes of *TA* genes were basically targeted by different miRNAs. Genes in class 1 had more potential miRNA (50 in total) than class 2 (32 in total), but genes in class 2 had more shared miRNA (18/32) than class 1 (17/50), which implied that genes in class 2 might be more conservative. Notably, there were four miRNAs (miR408, miR909, miR6021, and miR8678) that could target both two classes of genes.

### Expression Profiling of *TA* Genes in Vegetative and Reproductive Tissues

**FIGURE 6:**
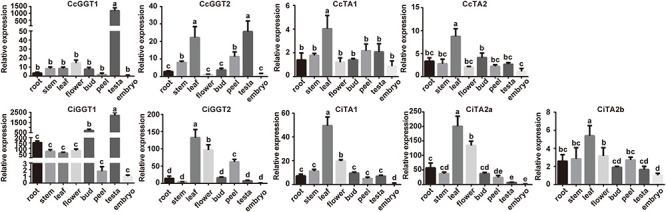
Expression analysis of *GGT* and *TA* genes in different tissues in Chinese hickory and pecan by RT-qPCR. The analysis was carried out using three biological replicates and three technical replicates for each sample. The error bars represented the standard deviations of nine replicates. Different letters indicated significant differences according to the Tukey–Kramer test (*P* < 0.05).

In order to investigate the expression profiles of *TA* genes, eight main tissues were collected for quantitative real-time PCR, including roots, stems, leaves, female flowers, buds, peels, testae (seed coats), and embryos. Since *GGT* is a key tannin pathway synthesis gene, we simultaneously quantified its expression pattern (Figure 6 and Supplementary Figure 4). The results showed that the abundance of *CcGGT1* in the seed coat was more than 100 times higher than in other tissues and *CcGGT2* was both highly expressed in seed coat and leaf. In pecan, *CiGGT1* had more than 2000 times higher expression in seed coat than embryo, followed by bud. On the contrary, the abundance of *CiGGT2* in leaf, flower, and peel was 50–150 times higher than in seed coat. These results suggest that *GGT1* was the main factor to determine the astringent taste in seed coat. *GGT2* was involved in the accumulation of tannin in the leaves in addition to the seed coat. This expression pattern suggested that *GGT2* played a key role in the resistance of leaves to insect feeding and more tannins may exist in bud and flower in pecan to enhance the response to the environment stress. Compared with the *GGT* genes with different expression patterns, the pattern of *TA* genes functioned as tannin acyl-hydrolase was much closer in Chinese hickory and pecan. All five *TA* genes had high expression in leaves, but low expression in seed coat. Taken together, these results showed that leaves and seed coat were the main tissues of tannin accumulation, and the diverse expression pattern of the synthesis-related gene *GGTs* and hydrolase gene *TAs* indicated their important roles in the regulation mechanism of tannins in different tissue.

### Role of *GGT* and *TA* Genes in Response to Wounding Treatments

**FIGURE 7:**
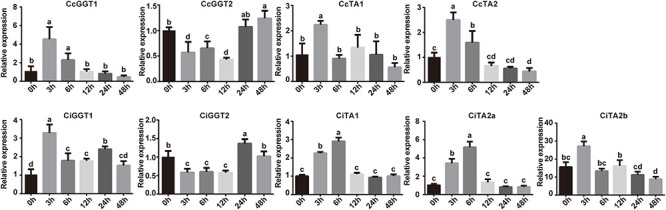
Expression analysis of *GGT* and *TA* genes induced by wound stress in Chinese hickory and pecan by RT-qPCR. The analysis was conducted based on three biological replicates and three technical replicates for each sample. The error bars represented the standard deviations of nine replicates. Different letters indicated significant differences according to the Tukey–Kramer test (*P* < 0.05).

According to the expression pattern of *GGT* and *TA* genes in different tissues (Figure 6), we found that *CcGGT2* and *CiGGT2* showed a relatively high expression in leaves. The expression level of *TAs* was also strongly up-regulated in leaves than in other tissues. In Chinese hickory, the abundance of *CcTAs* in leaves was only several times higher than other tissues, but in pecan, *CiTAs* were up to 200 times higher than other tissues. The high expression of two tannin-associated genes in leaves may be related to the resistance mechanism of plants to insect feeding (Barbehenn and Peter Constabel, 2011; Moctezuma et al., 2014). To provide insight into possible physiological roles of *TA* genes, we simulated insect herbivory under controlled conditions by leaf damage stress to investigate the expression of these genes. All *TA* and *GGT* genes in Chinese hickory and pecan were detected using RT-qPCR at 0, 3, 6, 12, 24, and 48 h after wound stress (Figure 7 and Supplementary Figure 5). When leaves were stressed by external damage, *CcGGT1* and *CiGGT1* quickly reached the maximum expression level in 3 h, and then immediately returned to normal expression. In contrast, *CcGGT2* and *CiGGT2* were down-regulated initially and returned to normal expression after 24 h. Five *TAs* shared the same expression pattern: rapidly reached the maximum expression in 3 or 6 h and then decreased to the normal level. Among them, *CiTA1* and *CiTA2a* were up-regulated slightly later and peaked in 6 h. This result suggested that Chinese hickory and pecan might own a rapid tannin-dependent defense mechanism to resistance to insect herbivory. Within 3 h after leaf injury, tannin synthetase and hydrolase genes were highly expressed to synthesize a series of tannin-related substances to resist insects. After 6 h, these genes returned to normal levels, which may mean the end of the defense process. Interestingly, the expression of *CcGGT2* and *CiGGT2* was completely opposite to *GGT1* genes. The expression of *GGT2* decreased in 3 h and increased after 24 h later. It is possible that *GGT2* does not participate in the defense against insects, or *GGT2* was only involved in the reconstruction of defense at the late stage (after 24 h) and this mechanism is worth revealing.

### Expression Pattern of *TA* Genes During Embryo Development in Seed Coats

The seed coat is the main tissue of astringency source in the edible part in Juglandaceae. The astringency gradually accumulated with the extension of the seed development period.

**FIGURE 8:**
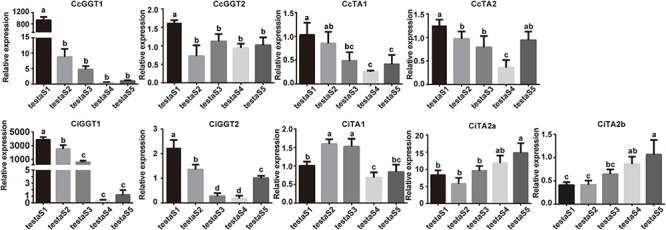
Expression analysis of *GGT* and *TA* genes in seed coats in Chinese hickory and pecan by RT-qPCR. The analysis was performed using three biological replicates and three technical replicates for each sample. The error bars represented the standard deviations of nine replicates. Different letters indicated significant differences according to the Tukey–Kramer test (*P* < 0.05).

The quantitative real-time PCR results of different developmental stages of the seed coat showed that both *GGT1* and *GGT2* were the highest expressions in the S1 stage in Chinese hickory and pecan (Figure 8). The expression change of *GGT1* was much higher than that of *GGT2*, which indicated that *GGT1* may be the most important gene that participated in tannin synthesis in the seed coat. The expression of *CiGGT1* was decreased 3,000-fold, while *CcGGT1* was decreased only 800-fold. On the contrary, the expressions of *CcTAs* and *CiTAs* did not show significant changes. *CcTA1* and *CcTA2* continued to down-regulate from the S1 to the S4 stage, and slightly increased in S5. Three *TA* genes in pecan showed two expression patterns. The expression level of *CiTA2a* and *CiTA2b* continued to increase, while *CiTA1* was lowly expressed in the S1 stage, up-regulated in S2 and S3, and then decreased. Taken together, the above results indicated that the expressions of the synthesis-related gene *GGTs* in two species had great influence in tannin accumulated especially in early stage of seed coat development, but the hydrolase gene *TA*s continued to hydrolyzed throughout the developmental period. The expression patterns of *GGT* genes may lead to the large accumulation of tannins in the early stage of seed coat development, accompanied by the expression of *TA* genes. However, at the maturity stage, the decrease of *GGT* expression resulted in tannins that were no longer synthesized in large quantities. At the same time, the stable expression of *TA* genes resulted in a continuous decrease in the accumulated tannin content. Moreover, compared with the down-regulation of both *CcTA* genes in Chinese hickory, two of three *CiTA* genes were up-regulated in the mature stage, which may further enhance the ability to hydrolyze tannins in pecan, resulting in the lighter astringency.

### Astringency Assessment in the Seed Coats of Chinese Hickory and Pecan

**FIGURE 9:**
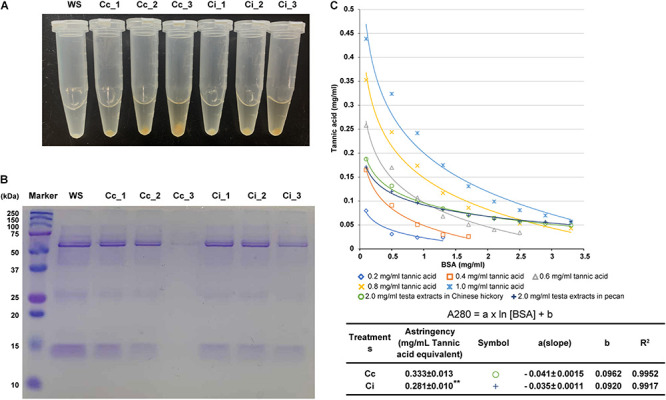
Astringency assessment in the seed coats of Chinese hickory and pecan. **(A)** The difference of precipitate binding by human salivary proteins and the astringent substance in seed coat extracts. WS, salivary protein profile obtained for whole saliva; Cc_1-Cc_3, the residual protein in the supernatant after reaction of saliva and the three concentrations (0.625, 1.25, and 2.5 mg/ml) of mature seed coat extracts in Chinese hickory; Ci_1-Ci_3, the residual protein in the supernatant after reaction of saliva and the three concentrations (0.625, 1.25, and 2.5 mg/ml) of mature seed coat extracts in pecan. **(B)** SDS-PAGE gel electrophoresis of human salivary proteins in the supernatant of reactions. **(C)** Influence of serum albumin (BSA) additions on A280 nm from different tannic acid solutions and seed coat extracts. Cc: seed coat extracts in Chinese hickory; Ci: seed coat extracts in pecan. Data were expressed as mean ± SD (*n* = 3). The asterisk stands for significant difference (*p* < 0.01) in astringency between Chinese hickory and pecan.

Furthermore, we detected the astringency in the mature seed coats between Chinese hickory and pecan with two assays. After incubating the seed coat extracts of the two species and human salivary proteins, the results of centrifugation in the bottom of tubes showed that obvious precipitation appeared at different concentrations of the extractions in two species compared with the control (Figure 9A). At the maximum concentration, the precipitation from seed coat extracts in Chinese hickory was obviously more than that in pecan. SDS-PAGE gel electrophoresis also showed that seed coat extracts in Chinese hickory had less salivary protein in the supernatant (Figure 9B), which proved that Chinese hickory had stronger astringency.

The other assay estimated the astringency by the precipitation of tannins, resulting in a decrease in the absorbance value at 280 nm, and the relationship between absorbance value and protein concentration was logarithmic (Llaudy et al., 2004; Jauregi et al., 2016). The slope of the logarithmic equation decreased with increasing tannins, and the calibration curve obtained by plotting the tannin concentration against the slope was linear with a regression coefficient of 0.997. We determined the slope of the logarithmic equation for the seed coat extracts in Chinese hickory and pecan and converted the astringency of the seed coat extracts to the tannic acid standard according to a linear equation. The result showed that the astringency of seed coat in Chinese hickory was 0.333, which was highly significantly greater than 0.281 in pecan (*p*-value = 0.005) (Figure 9C). All these two results confirmed our taste feeling that the seed coat of Chinese hickory was more astringent than pecan.

### The Phenolic Compounds in the Seed Coats of Chinese Hickory, Pecan, and Walnut

**FIGURE 10:**
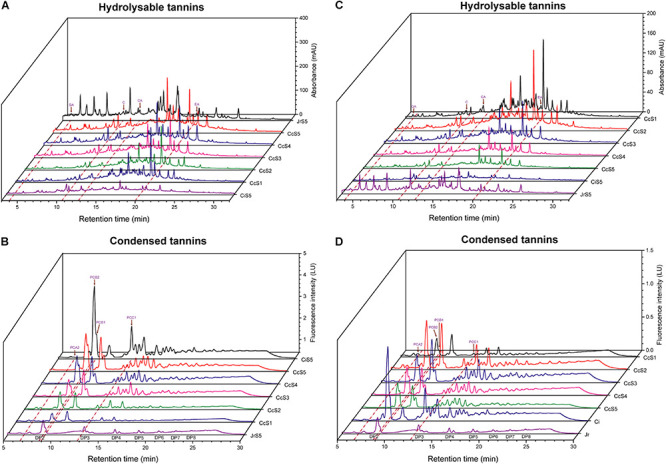
HPLC analysis of phenolic compounds in the seed coat of maturity stage in pecan (CiS5), walnut (JrS5), and Chinese hickory (CcS1-CcS5). **(A)** Low-molecular-weight phenolic compounds including hydrolyzable tannins in fresh sample; **(B)** condensed tannins in fresh sample; **(C)** low-molecular-weight phenolic compounds including hydrolysable tannins in dry sample; **(D)** condensed tannins in dry sample. GA, gallic acid; C, catechin; CA, caffeic acid; EA, ellagic acid; PCA2, procyanidin A2; PCB2, procyanidin B2; PCB1, procyanidin B1; PCC1, procyanidin C1.

To evaluate the content of astringent phenolic substances in the seed coat of Chinese hickory and pecan, we detected condensed tannins and other low-molecular-weight phenolic compounds (including hydrolyzable tannins, flavonoids, and phenolic acids) in the seed coats of mature seeds in three Juglandaceae species and in the different developmental stages of seed coats in Chinese hickory based on previous research methods (Gong and Pegg, 2017) (Figure 10). Comparing the other two species, the seed coats of pecan have the highest content of condensed tannins and the lowest content of phenolic compounds with low molecular weight, and the seed coats of walnut had the highest content of phenolic compounds and the lowest content of condensed tannins, while the content of two types of polyphenols in the seed coats of Chinese hickory was at the median level. With the ripening of seeds, the content of phenolic compounds with a low molecular weight in dry samples of seed coats was continuously decreased. The content of condensed tannin was the highest in the S2 period and was decreased from the S3 to S5 period. Notably, the water content of the seed coats varies greatly throughout the fruit ripening stage. Therefore, the trend in fresh seed coats was completely opposite, and the contents of hydrolyzable tannins and condensed tannins showed an upward trend, possibly leading to the increase of astringency in seed coats.

## Discussion

Tannins are an important plant polyphenol and have been classified into two main groups, condensed tannins (CT) and hydrolyzable tannins (HT). The plants in the Juglandaceae are rich in tannins, both CT and HT, and different species have a different component proportion. The plant tannase gene was first discovered in tea plants in 2020 (Dai et al., 2020) and was found to be widely distributed in many species rich in tannins. According to the blast results of *CsTA* in different plant species, *TA* genes and the neighboring clade of carboxylesterase genes (named *TA-like* genes) have very similar sequences compared with other clades (Supplementary Figure 1). In the phylogenetic tree of tannase genes, *TA, TA-like*, and acetate esterase genes all belonged to one clade. In *Arabidopsis*, although eight genes were acetate esterase genes, none of them belonged to the *TA* or *TA*-like clade. In contrast, persimmon has one *TA* gene, but no *TA*-like gene. This phenomenon may be related to the difference in tannin synthesis and degradation in different species. For example, Arabidopsis and rice mainly contain flavonoid-type condensed tannins, while there are no related reports that these two plants contained hydrolyzable tannins (Zhao et al., 2010; Goufo and Trindade, 2014; Shao and Bao, 2015; Demonsais et al., 2020). At the same time, these two species also lack the key genes *SDH* and *GGT* for hydrolyzable tannin synthesis as well as the *TA* genes responsible for the degradation of hydrolyzable tannins. Therefore, we speculate that the *TA* genes may be distributed in plants rich in hydrolyzable tannins.

### Tandem Repeats of *TA* Genes Indicated Their Evolutionary Origin

According to the genome distribution of *TA* and *TA*-like genes from all species we identified, we found that most of these genes were located in a very small region of a chromosome (Supplementary Figure 3 and Supplementary Table 6). These results showed that *TA* and *TA*-like genes were tandem repeat genes. In pecan, Chinese hickory, strawberry, and grape, all the *TA* and *TA*-like genes were located in a less than 100-kb region, and seven genes were in 15 kb in grape. In pomegranate, *TA* genes and *TA*-like genes were distributed on two different chromosomes. In walnut and tea, in addition to one gene, other genes are all located in the same region on the same chromosome. Furthermore, we investigated all the genes in these regions and found that all genes are *TA* genes or *TA-like* genes in Chinese hickory and pecan. In other species, most of the genes in the region belonged to the carboxylesterase gene. Tandem repeats of these genes suggested that tannins and tannase were very important for these species. During evolution, carboxylesterase genes produced multiple copies. Some of them kept their original function belonging to *CXE* genes, and others formed the function of tannase as paralogous genes that belong to *TA* genes through cumulative mutation. These results demonstrated a strong linkage between *CXEs* and *TAs*, which is difficult to separate during plant breeding and needs great attention.

Many metabolites with diversified chemical compounds in plants are produced by the replication, divergence, and selection of metabolic-related enzyme genes. Generally, the more types of metabolites, the more copy of genes are required. In different plants, there are big differences in the number of genes, like triterpenoids (Khakimov et al., 2015; Itkin et al., 2016; Erthmann et al., 2018; Cárdenas et al., 2019; Liu et al., 2019). Tandem repeat is the most important source in the formation of these genes. On the one hand, the copy number of *TA* genes produced by tandem repeats may affect the ability to hydrolyze tannins in different tissue and even different plants. On the other hand, analyzing the history of tandem repeat formation from the perspective of species evolution may be important for the study of tannin protection mechanisms in plants.

### High Expression of Tannase in Leaves May Effectively Resist Herbivores and Microbial Infection

Tannin usually refers to the chemical defense substances against herbivores, which are mainly divided into hydrolyzable tannins and condensation tannins (Boudet, 2007; Miranda et al., 2007; Thipyapong et al., 2007). Tannin mainly forms toxic semiquinone through oxidation and reduces the digestive enzyme activity of herbivores. However, the effects of the two types of tannins are different. Hydrolyzable tannins could resist herbivorous insects, but condensed tannins are ineffective under the condition of high pH (alkaline) in the intestinal tract of insects (Barbehenn et al., 2006; Barbehenn and Peter Constabel, 2011). Although condensed tannins do not affect herbivorous insects, they increase after leaf injury (Osier and Lindroth, 2004; Stevens et al., 2007). The expression of tannase can accumulate more ellagic acid in tissues, further forming ellagic tannins to resist herbivores such as insects. Furthermore, gallic acid produced by hydrolysis of hydrolyzable tannins (HTs) with tannase is an important component, which can effectively inhibit high expression of fungi like *Aspergillus flavus*, so that tissues have stronger antibacterial ability and reduce fungal infection (Mahoney and Molyneux, 2004).

Leaves are critical to photosynthesis and are the main tissues that plants need to protect. Although the total phenolic content in leaves is low, the main chemical defense substances—condensed tannins and hydrolyzable tannins—have a high proportion. In most plants, leaves are usually the highest tannin content in the whole plant (Barbehenn and Peter Constabel, 2011; Dettlaff et al., 2018). Gallardo et al. (2019) showed that the expression of tannin synthesis-related genes in *Quercus ilex* leaves increased after mechanical damage treatment, including condensed tannin synthesis-related enzymes like ANR, LAR, ANS, and SDH1, and hydrolyzable tannin synthesis-related enzyme SDH2. After mechanical damage treatment, the content of total phenol, total tannin, and condensed tannin all increased (Gallardo et al., 2019). Another research in *Stryphnodendron adstringens* also showed that the concentrations of condensed tannins and hydrolyzable tannins all increased, while total phenolics decreased after leaf clipping. Plants showed a trade-off between tannins and total phenols (Tuller et al., 2018). Our quantitative study showed that the expression of tannin-related genes *GGTs* and *TAs* in leaves of Chinese hickory and pecan was up-regulated rapidly after 3 h of abiotic stress and began to hydrolyze a large number of substances into small chemicals such as ellagic acid and gallic acid to resist wound stress. After 6 h, the resistance response gradually ended. This result provided a key time point for studying the abiotic stress in Chinese hickory and pecan, and a foundation for further research.

### *TA* Genes May Be Regulated by miRNA in Response to Plant Biotic and Abiotic Stresses

According to predicted miRNAs in walnut, pecan, and Chinese hickory, we found that the *TAs* could be targeted by many miRNAs. This meant that the regulation mechanism of tannase genes was much more complicated than we thought. Based on the targeted network of miRNAs and targeted *TAs* in three species, it was found that *TA* genes from class 1 and class 2 were very diverse and they are targeted by different miRNAs. So, it is likely that two classes of *TA* genes are involved in different biological processes, regulating the tannins by different regulatory pathways. Nevertheless, there are still four miRNAs that can regulate genes in class 1 and class 2 simultaneously. Like miR408, one of the most conserved plant miRNAs was report as a wound-related miRNA in sweet potato and was repressed by wounding and jasmonate (Kuo et al., 2019). It was proved that jasmonate could induce hydrolyzable tannins and participated in wound response in red oak (Allison and Schultz, 2004; Elderd et al., 2013). In *Arabidopsis*, miR408 participates in seed yield and abiotic stress such as salinity, cold, oxidative stress, drought, and osmotic stress (Ma et al., 2015; Song et al., 2018). In addition, it was proved to be involved in photosynthesis, growth, and other biological processes (Pan et al., 2018). miR6021 is a specific miRNA found in tobacco, which can regulate plant innate immune receptors and was predicted to target a CC-NB-LRR gene, *Hcr9* in *Solanaceae* (Li et al., 2012). In each class, there exists a lot of co-regulating miRNAs in different species. That implied that the conservation of regulation of two class *TA* genes in plants rich in tannins may be regulated by the same miRNAs. In addition to the miRNAs mentioned above, there are other miRNAs in the targeted network that are involved in response to biotic and abiotic stress.

### *TA* Genes Contributed Higher Astringency by Controlling Hydrolyzable Tannin Content in the Seed Coat of Chinese Hickory Than Pecans

Tannin is abundantly accumulated in seed coats in Juglandaceae, which is not only resistant to animal feeding and disease but also the main source of astringent taste when humans eat nuts. However, different plants have different tannin species preferences; for example, the fruits of grapes, persimmons, cocoa, and sorghum are dominated by condensed tannins (Zhu et al., 2019; Wei et al., 2020), while the fruits of pomegranates and walnuts contain more hydrolyzable tannins (Bajec and Pickering, 2008; Akhtar et al., 2015). Xu et al. (2020) carried out a comprehensive analysis of phenolic metabolites in eight tissues of pecan. The results showed that hydrolyzable tannins were the main phenolic metabolites in the seed coat of pecan. Hydrolyzable tannins have complex components and high content. Among them, the highest content in the seed coat is ellagic acid. Through HPLC separation, we found that the hydrolyzable tannin content in Chinese hickory was higher than that in pecan, while the condensed tannin content was lower than that in pecan. By our astringency assessment experiments, the seed coat of Chinese hickory is more astringent than pecan’s, indicating that hydrolyzable tannins may be the main source of astringency in the seed coat of Chinese hickory.

In addition, we found that the tannin content in fresh samples showed an upward trend during the seed coat development of Chinese hickory, which was consistent with our astringency assessment assay. However, after excluding the influence of water content change during seed coat development, the tannin content in dry sample decreased continuously. Real-time quantitative PCR results also confirmed that the expression of synthesis gene *GGTs* and hydrolysis gene *TA*s continued to decrease during the development of seed coat in Chinese hickory, resulting in corresponding changes in tannin content. At the same time, *CcTA*s are continuously down-regulated, while *CiTA2a* and *CiTA2b* are up-regulated, which may cause a significant difference in tannin content between Chinese hickory and pecan nuts at maturity. Thus, the various expression pattern of *TA* genes in Chinese hickory and pecan may lead to the difference in the content of hydrolyzable tannins, which are the main source of astringency in the seed coat at the maturity stage.

## Conclusion

Tannins are a large class of important metabolites of plants with a lot of structures. In 2020, the first plant tannase gene (*TA*) was discovered in tea (Dai et al., 2020). Tea mainly contains condensed tannins, while the seed coats of Juglandaceae are mainly composed of hydrolyzable tannins. Moreover, although they have similar genetic backgrounds, Chinese hickory and pecan have different levels of astringency in the seed coats. We identified and analyzed two and three tannase genes in Chinese hickory and pecan, respectively. The expression of *GGT* and *TA* genes in seed coat during seed development showed that the diversity patterns implied different mechanisms in tannin metabolism. Phenolic compounds separated by HPLC in the seed coats showed that hydrolyzable tannin content in Chinese hickory was higher than that in pecan, while the condensed tannin content was lower than that in pecan. This suggests that the content of hydrolyzed tannin is the main reason for the difference in astringency between the two species. Taken together, the early stage of seed development is a critical period for tannin metabolism in seed coats. Due to the continuous expression of *TA* genes at the maturity stage, the tannin content in the seed coats decreases, but the percentage of tannins is increased due to the decrease of water content during the maturation process. At the later stages of development, the differential change of expression level in *TA* genes in Chinese hickory and pecan may be the source of the final difference in astringency between the two species.

## Data Availability Statement

The datasets generated for this study can be found in online repositories. The names of the repository/repositories and accession number(s) can be found in the article/Supplementary Material.

## Author Contributions

KW, YL, and JHu conceived and designed this study. KW and JW analyzed the data and wrote the manuscript. JW and SL performed the experiments. All authors have read and approved this manuscript.

## Conflict of Interest

The authors declare that the research was conducted in the absence of any commercial or financial relationships that could be construed as a potential conflict of interest.

## Footnotes

1 http://gigadb.org/dataset/100571

2 http://xhhuanglab.cn/data/juglans.html

3 http://pfam.xfam.org/

4 http://smart.embl-heidelberg.de/

5 http://web.expasy.org/protparam/

6 http://www.cbs.dtu.dk/services/SignalP-4.0/

7 http://www.cbs.dtu.dk/services/TMHMM/

8 https://mafft.cbrc.jp/alignment/server/

9 http://meme-suite.org/tools/meme

10 http://bioinformatics.psb.ugent.be/webtools/plantcare/html/

11 https://www.dna.affrc.go.jp/PLACE/?action=newplace

12 http://plantgrn.noble.org/psRNATarget/

13 https://www.ncbi.nlm.nih.gov/tools/primer-blast/

## Supplementary Material

Supplementary Figure 1 Multiple sequence alignment of TA and TA-like proteins. The alignment was constructed by MAGA and visualized by GENEDOC. The blue box represented the position of the reported conserved domain of tannase (Dai et al., 2020). Red line marked the location of motif 7.

Supplementary Figure 2 Prediction of *cis*-acting elements in *TA* gene promoter regions. All *cis*-acting elements were marked with different colors according to the potential biological process.

Supplementary Figure 3 Scaffold locations and tandem repeat of *TA* genes in plants. The *TA* and *TA-like* genes were marked with red. Scaffold numbers are shown on the left side of each respective scaffold. The different genes are located according to their genomic position.

Supplementary Table 1 Summary of *TA* genes and other carboxylesterases.

Supplementary Table 2 Detailed information of plant TA and TA-like proteins.

Supplementary Table 3 Summary of conserved domain in TA and TA-like proteins in Juglandaceae.

Supplementary Table 4 Potential miRNAs and target *TA* genes in Juglandaceae.

Supplementary Table 5 The list of primers sequence used for RT-qPCR.

Supplementary Table 6 Scaffold location of *TA* and *TA-like* genes in plants.

## References

[B1] Abdel-Naby M. A. El-Tanash A. B. Sherief A. D. A. (2016). Structural characterization, catalytic, kinetic and thermodynamic properties of Aspergillus oryzae tannase. *Int. J. Biol. Macromol.* 92 803–811. 10.1016/j.ijbiomac.2016.06.098 27373426

[B2] Akhtar S. Ismail T. Fraternale D. Sestili P. (2015). Pomegranate peel and peel extracts: chemistry and food features. *Food Chem.* 174 417–425. 10.1016/j.foodchem.2014.11.035 25529700

[B3] Allison S. D. Schultz J. C. (2004). Differential activity of peroxidase isozymes in response to wounding, gypsy moth, and plant hormones in northern red oak (Quercus rubra L.). *J. Chem. Ecol.* 30 1363–1379. 10.1023/b:joec.0000037745.66972.3e 15503525

[B4] Bailey T. L. Boden M. Buske F. A. Frith M. Grant C. E. Clementi L. (2009). MEME SUITE: tools for motif discovery and searching. *Nucleic Acids Res.* 37 W202–W208. 10.1093/nar/gkp335 19458158 PMC2703892

[B5] Bajec M. R. Pickering G. J. (2008). Astringency: mechanisms and perception. *Crit. Rev. Food Sci. Nutr.* 48 858–875. 10.1080/10408390701724223 18788010

[B6] Barbehenn R. V. Jones C. P. Hagerman A. E. Karonen M. Salminen J.-P. (2006). Ellagitannins have greater oxidative activities than condensed tannins and galloyl glucoses at high pH: potential impact on caterpillars. *J. Chem. Ecol.* 32 2253–2267. 10.1007/s10886-006-9143-7 17019621

[B7] Barbehenn R. V. Peter Constabel C. (2011). Tannins in plant-herbivore interactions. *Phytochemistry* 72 1551–1565. 10.1016/j.phytochem.2011.01.040 21354580

[B8] Boudet A.-M. (2007). Evolution and current status of research in phenolic compounds. *Phytochemistry* 68 2722–2735. 10.1016/j.phytochem.2007.06.012 17643453

[B9] Cammann J. Denzel K. Schilling G. Gross G. G. (1989). Biosynthesis of gallotannins: β-glucogallin-dependent formation of 1,2,3,4,6-pentagalloylglucose by enzymatic galloylation of 1,2,3,6-tetragalloylglucose. *Arch. Biochem. Biophys.* 273 58–63. 10.1016/0003-9861(89)90161-6 2757399

[B10] Cárdenas P. D. Almeida A. Bak S. (2019). Evolution of structural diversity of triterpenoids. *Front. Plant Sci.* 10:1523. 10.3389/fpls.2019.01523 31921225 PMC6929605

[B11] Chen C. Chen H. Zhang Y. Thomas H. R. Frank M. H. He Y. (2020). TBtools: an integrative toolkit developed for interactive analyses of big biological data. *Mol. Plant* 13 1194–1202. 10.1016/j.molp.2020.06.009 32585190

[B12] Combs C. A. (2016). *Tannins: Biochemistry, Food Sources and Nutritional Properties.* Hauppauge, NY: Nova Science Publishers.

[B13] Dai X. Liu Y. Zhuang J. Yao S. Liu L. Jiang X. (2020). Discovery and characterization of tannase genes in plants: roles in hydrolysis of tannins. *New Phytol.* 226 1104–1116. 10.1111/nph.16425 32061142

[B14] Dai X. Zhao P. X. (2011). psRNATarget: a plant small RNA target analysis server. *Nucleic Acids Res.* 39 W155–W159. 10.1093/nar/gkr319 21622958 PMC3125753

[B15] de Jesus N. Z. T. de Souza Falcão H. Gomes I. F. de Almeida Leite T. J. de Morais Lima G. R. Barbosa-Filho J. M. (2012). Tannins, peptic ulcers and related mechanisms. *Int. J. Mol. Sci.* 13 3203–3228. 10.3390/ijms13033203 22489149 PMC3317710

[B16] Demonsais L. Utz-Pugin A. Loubéry S. Lopez-Molina L. (2020). Identification of tannic cell walls at the outer surface of the endosperm upon Arabidopsis seed coat rupture. *Plant J.* 104 567–580. 10.1111/tpj.14994 32985026 PMC7702108

[B17] Denzel K. Gross G. G. (1991). Biosynthesis of gallotannins. *Planta* 184 285–289. 10.1007/BF00197959 24194082

[B18] Dettlaff M. A. Marshall V. Erbilgin N. Cahill J. F. (2018). Root condensed tannins vary over time, but are unrelated to leaf tannins. *AoB Plants* 10 ly044. 10.1093/aobpla/ply044 30090221 PMC6070047

[B19] Elderd B. D. Rehill B. J. Haynes K. J. Dwyer G. (2013). Induced plant defenses, host-pathogen interactions, and forest insect outbreaks. *Proc. Natl. Acad. Sci. U. S. A.* 110 14978–14983. 10.1073/pnas.1300759110 23966566 PMC3773759

[B20] Erthmann P. Ø Agerbirk N. Bak S. (2018). A tandem array of UDP-glycosyltransferases from the UGT73C subfamily glycosylate sapogenins, forming a spectrum of mono- and bisdesmosidic saponins. *Plant Mol. Biol.* 97 37–55. 10.1007/s11103-018-0723-z 29603041

[B21] Finn R. D. Coggill P. Eberhardt R. Y. Eddy S. R. Mistry J. Mitchell A. L. (2016). The Pfam protein families database: towards a more sustainable future. *Nucleic Acids Res.* 44 D279–D285. 10.1093/nar/gkv1344 26673716 PMC4702930

[B22] Gallardo A. Morcuende D. Solla A. Moreno G. Pulido F. Quesada A. (2019). Regulation by biotic stress of tannins biosynthesis in Quercus ilex: crosstalk between defoliation and Phytophthora cinnamomi infection. *Physiologia Plantarum* 165 319–329. 10.1111/ppl.12848 30294855

[B23] Gasteiger E. (2003). ExPASy: the proteomics server for in-depth protein knowledge and analysis. *Nucleic Acids Res.* 31 3784–3788. 10.1093/nar/gkg563 12824418 PMC168970

[B24] Gong Y. Pegg R. B. (2017). Separation of ellagitannin-rich phenolics from U.S. pecans and Chinese hickory nuts using fused-core HPLC columns and their characterization. *J. Agric. Food Chem.* 65 5810–5820. 10.1021/acs.jafc.7b01597 28648053

[B25] Goufo P. Trindade H. (2014). Rice antioxidants: phenolic acids, flavonoids, anthocyanins, proanthocyanidins, tocopherols, tocotrienols, γ-oryzanol, and phytic acid. *Food Sci. Nutr.* 2 75–104. 10.1002/fsn3.86 24804068 PMC3959956

[B26] Guo W. Chen J. Li J. Huang J. Wang Z. Lim K. J. (2020). Portal of juglandaceae: a comprehensive platform for juglandaceae study. *Hortic. Res.* 7:35. 10.1038/s41438-020-0256-x 32194971 PMC7072074

[B27] Higo K. Ugawa Y. Iwamoto M. Korenaga T. (1999). Plant cis-acting regulatory DNA elements (PLACE) database: 1999. *Nucleic Acids Res.* 27 297–300. 10.1093/nar/27.1.297 9847208 PMC148163

[B28] Huang Y. Xiao L. Zhang Z. Zhang R. Wang Z. Huang C. (2019). The genomes of pecan and Chinese hickory provide insights into Carya evolution and nut nutrition. *GigaScience* 8:giz036. 10.1093/gigascience/giz036 31049561 PMC6497033

[B29] Itkin M. Davidovich-Rikanati R. Cohen S. Portnoy V. Doron-Faigenboim A. Oren E. (2016). The biosynthetic pathway of the nonsugar, high-intensity sweetener mogroside V from Siraitia grosvenorii. *Proc. Natl. Acad. Sci. U. S. A.* 113 E7619–E7628. 10.1073/pnas.1604828113 27821754 PMC5127336

[B30] Jahanban-Esfahlan A. Ostadrahimi A. Tabibiazar M. Amarowicz R. (2019). A comparative review on the extraction, antioxidant content and antioxidant potential of different parts of walnut (juglans regia l.) fruit and tree. *Molecules* 24:2133. 10.3390/molecules24112133 31195762 PMC6600437

[B31] Jana A. Halder S. K. Banerjee A. Paul T. Pati B. R. Mondal K. C. (2014). Biosynthesis, structural architecture and biotechnological potential of bacterial tannase: a molecular advancement. *Bioresour. Technol.* 157 327–340. 10.1016/j.biortech.2014.02.017 24613317

[B32] Jauregi P. Olatujoye J. B. Cabezudo I. Frazier R. A. Gordon M. H. (2016). Astringency reduction in red wine by whey proteins. *Food Chem.* 199 547–555. 10.1016/j.foodchem.2015.12.052 26776007

[B33] Jia X. Luo H. Xu M. Zhai M. Guo Z. Qiao Y. (2018). Dynamic changes in phenolics and antioxidant capacity during pecan (*Carya illinoinensis*) kernel ripening and its phenolics profiles. *Molecules* 23:435. 10.3390/molecules23020435 29462910 PMC6017656

[B34] Khakimov B. Kuzina V. Erthmann P. Ø Fukushima E. O. Augustin J. M. Olsen C. E. (2015). Identification and genome organization of saponin pathway genes from a wild crucifer, and their use for transient production of saponins in Nicotiana benthamiana. *Plant J.* 84 478–490. 10.1111/tpj.13012 26333142

[B35] Krogh A. Larsson B. von Heijne G. Sonnhammer E. L. L. (2001). Predicting transmembrane protein topology with a hidden markov model: application to complete genomes11Edited by F. Cohen. *J. Mol. Biol.* 305 567–580. 10.1006/jmbi.2000.4315 11152613

[B36] Kuo Y. W. Lin J. S. Li Y. C. Jhu M. Y. King Y. C. Jeng S. T. (2019). MicroR408 regulates defense response upon wounding in sweet potato. *J. Exp. Bot.* 70 469–483. 10.1093/jxb/ery381 30403812 PMC6322576

[B37] Lamy E. Pinheiro C. Rodrigues L. Capela e Silva F. Lopes O. S. Tavares S. (2016). “*Determinants of tannin-rich food and beverage consumption: oral perception vs. psychosocial aspects,” in Tannins: Biochemistry, Food Sources and Nutritional Properties*, ed. Combs C. A. (Hauppauge, NY: Nova Science Publishers), 29–58.

[B38] Lekha P. K. Lonsane B. K. (1997). Production and application of tannin acyl hydrolase: state of the art. *Adv. Appl. Microbiol.* 44 215–260. 10.1016/S0065-2164(08)70463-5. 9311108

[B39] Lescot M. (2002). PlantCARE, a database of plant cis-acting regulatory elements and a portal to tools for in silico analysis of promoter sequences. *Nucleic Acids Res.* 30 325–327. 10.1093/nar/30.1.325 11752327 PMC99092

[B40] Letunic I. Doerks T. Bork P. (2012). SMART 7: recent updates to the protein domain annotation resource. *Nucleic Acids Res* 40 D302–D305. 10.1093/nar/gkr931 22053084 PMC3245027

[B41] Li F. Pignatta D. Bendix C. Brunkard J. O. Cohn M. M. Tung J. (2012). MicroRNA regulation of plant innate immune receptors. *Proc. Natl. Acad. Sci. U. S. A.* 109 1790–1795. 10.1073/pnas.1118282109 22307647 PMC3277104

[B42] Liu Q. Khakimov B. Cárdenas P. D. Cozzi F. Olsen C. E. Jensen K. R. (2019). The cytochrome P450 CYP72A552 is key to production of hederagenin-based saponins that mediate plant defense against herbivores. *New Phytol.* 222 1599–1609. 10.1111/nph.15689 30661245

[B43] Livak K. J. Schmittgen T. D. (2001). Analysis of relative gene expression data using real-time quantitative PCR and the 2-ΔΔCT method. *Methods* 25 402–408. 10.1006/meth.2001.1262 11846609

[B44] Llaudy M. C. Canals R. Canals J.-M. Rozés N. Arola L. Zamora F. (2004). New method for evaluating astringency in red wine. *J. Agric. Food Chem.* 52 742–746. 10.1021/jf034795f 14969525

[B45] Ma C. Burd S. Lers A. (2015). miR408 is involved in abiotic stress responses in Arabidopsis. *Plant J.* 84 169–187. 10.1111/tpj.12999 26312768

[B46] Mahoney N. Molyneux R. J. (2004). Phytochemical inhibition of aflatoxigenicity in aspergillus flavus by constituents of walnut (*Juglans regia*). *J. Agric. Food Chem.* 52 1882–1889. 10.1021/jf030812p 15053524

[B47] Miranda M. Ralph S. G. Mellway R. White R. Heath M. C. Bohlmann J. (2007). The transcriptional response of hybrid poplar (Populus trichocarpa x P. deltoides) to infection by Melampsora medusae leaf rust involves induction of flavonoid pathway genes leading to the accumulation of proanthocyanidins. *Mol. Plant Microbe Interact.* 20 816–831. 10.1094/MPMI-20-7-0816 17601169

[B48] Moctezuma C. Hammerbacher A. Heil M. Gershenzon J. Méndez-Alonzo R. Oyama K. (2014). Specific polyphenols and tannins are associated with defense against insect herbivores in the tropical oak *Quercus oleoides*. *J. Chem. Ecol.* 40 458–467. 10.1007/s10886-014-0431-3 24809533

[B49] Nakabayashi R. Yonekura-Sakakibara K. Urano K. Suzuki M. Yamada Y. Nishizawa T. (2014). Enhancement of oxidative and drought tolerance in Arabidopsis by overaccumulation of antioxidant flavonoids. *Plant J.* 77 367–379. 10.1111/tpj.12388 24274116 PMC4282528

[B50] Nakamura T. Yamada K. D. Tomii K. Katoh K. (2018). Parallelization of MAFFT for large-scale multiple sequence alignments. *Bioinformatics* 34 2490–2492. 10.1093/bioinformatics/bty121 29506019 PMC6041967

[B51] Niehaus J. U. Gross G. G. (1997). A gallotannin degrading esterase from leaves of pedunculate oak. *Phytochemistry* 45 1555–1560. 10.1016/S0031-9422(97)00261-6

[B52] Osier T. L. Lindroth R. L. (2004). Long-term effects of defoliation on quaking aspen in relation to genotype and nutrient availability: plant growth, phytochemistry and insect performance. *Oecologia* 139 55–65. 10.1007/s00442-003-1481-3 14740291

[B53] Pan J. Huang D. Guo Z. Kuang Z. Zhang H. Xie X. (2018). Overexpression of microRNA408 enhances photosynthesis, growth, and seed yield in diverse plants. *J. Integr. Plant Biol.* 60 323–340. 10.1111/jipb.12634 29330900

[B54] Petersen T. N. Brunak S. von Heijne G. Nielsen H. (2011). SignalP 4.0: discriminating signal peptides from transmembrane regions. *Nat. Methods* 8 785–786. 10.1038/nmeth.1701 21959131

[B55] Ramos-Pineda A. M. Carpenter G. H. García-Estévez I. Escribano-Bailón M. T. (2020). Influence of chemical species on polyphenol-protein interactions related to wine astringency. *J. Agric. Food Chem.* 68 2948–2954. 10.1021/acs.jafc.9b00527 30854856

[B56] Regueiro J. Sánchez-González C. Vallverdú-Queralt A. Simal-Gándara J. Lamuela-Raventós R. Izquierdo-Pulido M. (2014). Comprehensive identification of walnut polyphenols by liquid chromatography coupled to linear ion trap-Orbitrap mass spectrometry. *Food Chem.* 152 340–348. 10.1016/j.foodchem.2013.11.158 24444946

[B57] Ren B. Wu M. Wang Q. Peng X. Wen H. McKinstry W. J. (2013). Crystal structure of tannase from *Lactobacillus plantarum*. *J. Mol. Biol.* 425 2737–2751. 10.1016/j.jmb.2013.04.032 23648840

[B58] Salminen J.-P. (2018). Two-dimensional tannin fingerprints by liquid chromatography tandem mass spectrometry offer a new dimension to plant tannin analyses and help to visualize the tannin diversity in plants. *J. Agric. Food Chem.* 66 9162–9171. 10.1021/acs.jafc.8b02115 30136834 PMC6203188

[B59] Shannon P. (2003). Cytoscape: a software environment for integrated models of biomolecular interaction networks. *Genome Res.* 13 2498–2504. 10.1101/gr.1239303 14597658 PMC403769

[B60] Shao Y. Bao J. (2015). Polyphenols in whole rice grain: genetic diversity and health benefits. *Food Chem.* 180 86–97. 10.1016/j.foodchem.2015.02.027 25766805

[B61] Song Z. Zhang L. Wang Y. Li H. Li S. Zhao H. (2018). Constitutive expression of mir408 improves biomass and seed yield in arabidopsis. *Front. Plant Sci.* 8:2114. 10.3389/fpls.2017.02114 29422907 PMC5789609

[B62] Stevens M. T. Waller D. M. Lindroth R. L. (2007). Resistance and tolerance in Populus tremuloides: genetic variation, costs, and environmental dependency. *Evol. Ecol.* 21 829–847. 10.1007/s10682-006-9154-4

[B63] Tamura K. Peterson D. Peterson N. Stecher G. Nei M. Kumar S. (2011). MEGA5: molecular evolutionary genetics analysis using maximum likelihood, evolutionary distance, and maximum parsimony methods. *Mol. Biol. Evol.* 28 2731–2739. 10.1093/molbev/msr121 21546353 PMC3203626

[B64] Thipyapong P. Stout M. J. Attajarusit J. (2007). Functional analysis of polyphenol oxidases by antisense/sense technology. *Molecules* 12 1569–1595. 10.3390/12081569 17960074 PMC6149088

[B65] Treutter D. (2006). Significance of flavonoids in plant resistance: a review. *Environ. Chem. Lett.* 4 147–157. 10.1007/s10311-006-0068-8

[B66] Tuller J. Marquis R. J. Andrade S. M. M. Monteiro A. B. Faria L. D. B. (2018). Trade-offs between growth, reproduction and defense in response to resource availability manipulations. *PLoS One* 13:e0201873. 10.1371/journal.pone.0201873 30133458 PMC6104975

[B67] Vanholme R. Cesarino I. Rataj K. Xiao Y. Sundin L. Goeminne G. (2013). Caffeoyl Shikimate Esterase (CSE) is an enzyme in the lignin biosynthetic pathway in Arabidopsis. *Science* 341 1103–1106. 10.1126/science.1241602 23950498

[B68] Wei X. Ju Y. Ma T. Zhang J. Fang Y. Sun X. (2020). New perspectives on the biosynthesis, transportation, astringency perception and detection methods of grape proanthocyanidins. *Crit. Rev. Food Sci. Nutr.* 10.1080/10408398.2020.1777527 [Epub ahead of print] 32551848

[B69] Weisemann S. Denzel K. Schilling G. Gross G. G. (1988). Enzymatic synthesis of 1-O-phenylcarboxyl-β-d-glucose esters. *Bioorg. Chem.* 16 29–37. 10.1016/0045-2068(88)90035-1

[B70] Xu M. Liu P. Jia X. Zhai M. Zhou S. Wu B. (2020). Metabolic profiling revealed the organ-specific distribution differences of tannins and flavonols in pecan. *Food Sci. Nutr.* 8 4987–5006. 10.1002/fsn3.1797 32994960 PMC7500802

[B71] Yang Y. Xu R. Ma C. J. Vlot A. C. Klessig D. F. Pichersky E. (2008). Inactive methyl indole-3-acetic acid ester can be hydrolyzed and activated by several esterases belonging to the AtMES esterase family of arabidopsis. *Plant Physiol.* 147 1034–1045. 10.1104/pp.108.118224 18467465 PMC2442527

[B72] Zhang L.-L. Li J. Wang Y.-L. Liu S. Wang Z.-P. Yu X.-J. (2019). Integrated approaches to reveal genes crucial for tannin degradation in *Aureobasidium melanogenum* T9. *Biomolecules* 9:439. 10.3390/biom9090439 31480670 PMC6769594

[B73] Zhao J. Pang Y. Dixon R. A. (2010). The mysteries of proanthocyanidin transport and polymerization. *Plant Physiol.* 153 437–443. 10.1104/pp.110.155432 20388668 PMC2879784

[B74] Zhu Q. Xu Y. Yang Y. Guan C. Zhang Q. Huang J. (2019). The persimmon (*Diospyros oleifera Cheng*) genome provides new insights into the inheritance of astringency and ancestral evolution. *Hortic. Res.* 6:138. 10.1038/s41438-019-0227-2 31871686 PMC6917749

