# Supplementary material for: Genome-Wide Identification of Tannase Genes and Their Function of Wound Response and Astringent Substances Accumulation in Juglandaceae
Source: Front Plant Sci. 2021 May 17;12:664470. doi: 10.3389/fpls.2021.664470 (PMC8165273; doi:10.3389/fpls.2021.664470)
Supplement: Supplementary Figure 1 — Multiple sequence alignment of TA and TA-like proteins. The alignment was constructed by MAGA and visualized by GENEDOC. The blue box represented the position of the reported conserved domain of tannase (Dai et al., 2020). Red line marked the location of motif 7. [file Data_Sheet_1.pdf]

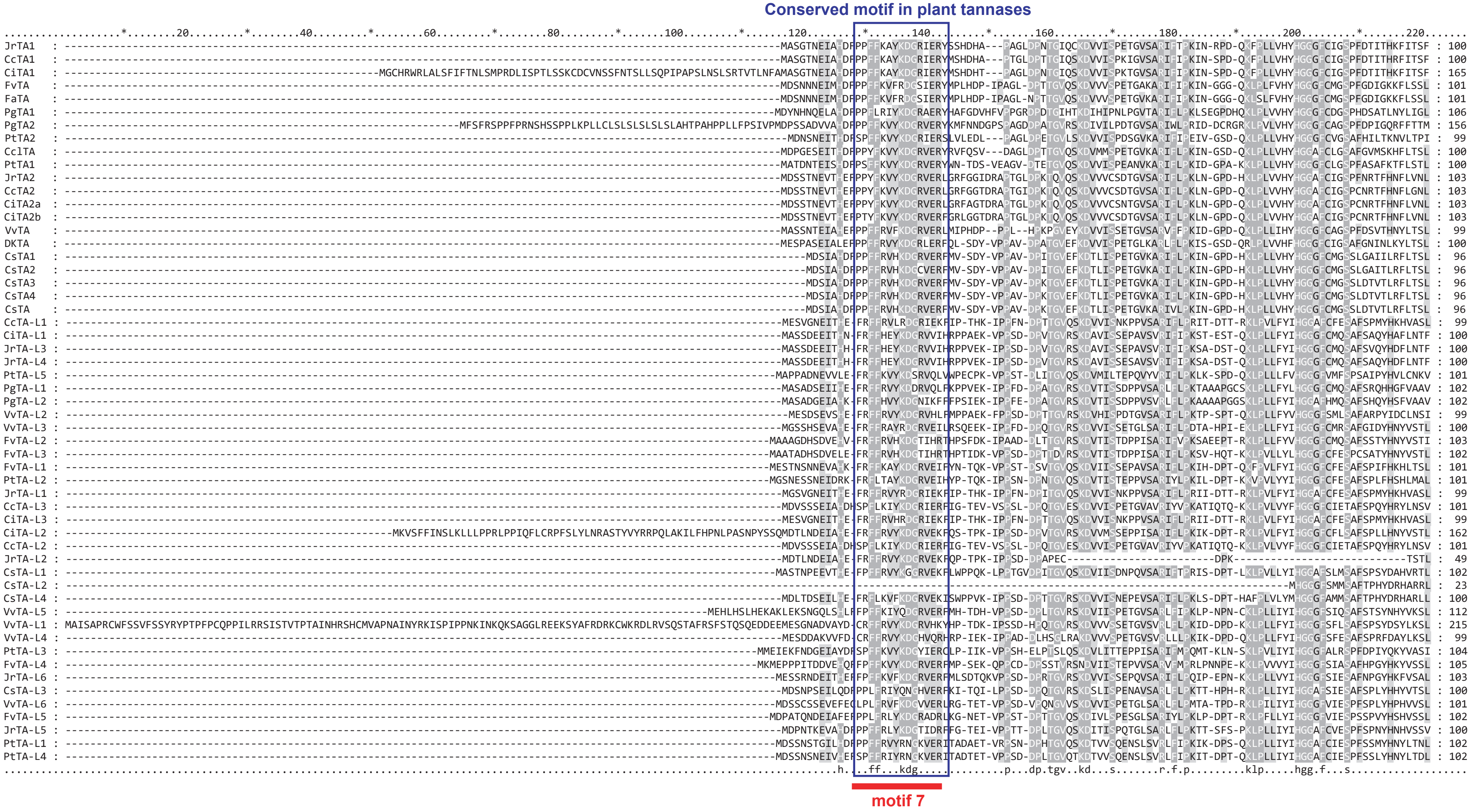
Conserved motif in plant tannases
motif 7

```
        .................*.......240.........*.......260.........*.......280.........*.......300.........*.......320.........*.......340.........*.......360.........*.......380.........*.......400.........*.......420.........*.......440........
JrTA1   : ILQANVIVVSVDYRLAPEHPLPIAYDDSWAALQWIAAHSNTQGPE--PWLNEYADFGRVFLTGESAGANISHHVAVRAGAA-GL---AGLKIIGVLIVHPFFGG-KDR--------------D---DMYKFLSSTSSGCSDDPKLNPAVD--PDLSKIGCAKVLVCVAEKDWLKDRGVTYSETLRKSG--------WG--------GGVELIE-------- : 271
CcTA1   : ILKANVIVVSVDYRLAPEHPLPIAYDDSWAALQWIAAHSNNQGPE--PWLNEYVDFGRVFLTGESAGANISHHVAVRAGAT-GL---AGLKIIGMLIVHPFFVG-KDR--------------D---EMYKFLSSTSSGCSDDPKLNPAVD--PDLSKMGCAKVLVCVAEKDRLKDRGVTYSETLRKSG--------WG--------GGVELIE-------- : 271
CiTA1   : ILQANVIVVSVDYRLAPEHPLPIAYDDSWEALQWIAAHSNNQGPE--PWLNEYADLGRVFLTGESAGANIGHYVAVRAGAT-GL---AGLKIIGMLIVHPFFVG-KDR--------------D---EMYKFLSSTSSGCSDDPKLNPAVD--PDLSKMGCAKVLVCVAEKDWLKDRGVTYWETLRKSD--------WG--------GGVELIE-------- : 336
FvTA    : VLKANVIVISVGYRLAPEHRLPIAYDDSASALHWISCHSNGQGPE--AWLNEYADLNRVFLGGESAGANIAHYVAVQAGVT-VP---LGLNIVGLVMVHPFFGG-KER--------------D---EMYKFLSSTSSGCDDDPKLNPAAD--PDLVKMPCGKVLVCVAEKDWLRDRGVSYHETLENLK-------GE---------GFALYE-------- : 272
FaTA    : VLKANVIVISVDYRLAPEHRLPIAYDDSSAALHWISCHSNGQGPE--AWLNEYADLNRVFLGGESAGANIAHYVAVQAGVT-VP---LGLNIVGLVMVHPFFGG-KEC--------------D---EMYKFLSSTSSGCDDDPKLNPAAD--PDLVKMPCGKVLVCVAEKDWLRDRGVSYHETLQNLK-------GE---------GFALYE-------- : 272
PgTA1   : VLGARVVAISIGYRLAPENPLPTAYEDSLEALKWLAS---GTEPD--PWVSQYADLGRVFLAGDSAGANIAHFVAVQAGIS-RLTVETGFKINGLLILHPFFGS-EEI------------SQN---QLYSYLYPASPGYENDPKLNPSVD--PDLHRIPVGRILICVGERDWIKDRGLAYYETLCKSE--------WS--------GTVELEE-------- : 279
PgTA2   : VPKANVIAIDVDYRLGPENPLPTAHEDSSEALKWLASHSGGHGPE--PWINEHADLNRVFLIGESAGANIAHYVAVRAGST-GL---TGLKIVGSIIAHPFFGG-KEP--------------D---KMIMFMYPGSPGTDEDPLLNPAVD--PDLPKMAGERVLVCVAENDWLMPRGVKYYETLRDGP--------WK--------GEVELDE-------- : 327
PtTA2   : VSRGNVIAISIDYRLAPEHLLPIAYNDSWDGLEWIAGHSNGLGPE--PWLNNHVDFGKVFLTGESAGANIAHYLAVQVGAN-GW---AGLKLAGVILVHPFFGY-KDV--------------D---EMHKYLCPTSSGGDDDPRLNPAVD--PNLSKMGCQKALVCVAEKDFLRDRGEAYYKTLATSG--------WP--------GKVEFYE-------- : 270
CclTA   : VSQANIIAISVDYRLAPEHPLPIAYDDSWAGLQWVAAHSNGLGPE--PWLNDHTDLGRVFLAGESAGANIAHYVAVQAGAT-KL---ASIKIHGLLNVHPFFGA-KEP--------------D---EMYKYLCPGSSGSDDDPKLNPAAD--PNLKNMAGDRVLVCVAEKDGLRNRGVAYYETLAKSE--------WD--------GHVEFYE-------- : 271
PtTA1   : ATQANVIAVSIDYRLAPEHKLPTAYDDSLAGLRWIAEHSDGKGPE--PWINEHADLGRVILAGESAGGTLAHYVAVQAGAA-GL---GGVAIKRLLIVHPYFGA-KEP--------------D---KFYQYMCPTSSGTDDDPKLNPAVD--PDLRLKGCDAVLVCVAEKDMLKGRGLAYYGAMKKSG--------WG--------GTVDLHE-------- : 271
JrTA2   : ASEANAVIISVDYRLAPEHLLPIAHEDSWAALQWIANHSKGQGAE--PWLNEHADFGRVFLAGESAGANIAHYVAVQAGAT-GL---AGPNIVGTLILHPYFGSKNGE--------------D---NLYKYMCPTSTGF-DDPLLYPEVD--PNLGRMAGGRVLVCVAENDWLKDRGLGYCETLRKNI--------WN--------GTVELYE-------- : 274
CcTA2   : ASEANAVIISVDYRLAPEHLLPIAHDDSWAALQWIANHSEGQGPE--PWLNEHADFGRVFLAGESAGANIAQYVAVQAGAT-GL---AGPNIVGTLILHPYFGSKNSE--------------D---KMYKYMCPTSTGF-DDPLLYPEVD--PNLGRMAGGRVLVCVAEKDWLKDRGLGYCETLRKSS--------WA--------GTVELYE-------- : 274
CiTA2a  : ASEANAVIISVDYRLAPEHLLPIAHEDSWAALQWIANHSEGQGPE--PWLNEHADFGRVFLAGESAGANIAQYVAVQAGAT-GL---AGPNIVGTLILHPYFGSKNSD--------------D---KMYKYMCPTSTGF-EDPLLYPEVD--PNLGRMAGGRVLVCVAEKDRLKDRGLGYCETLRQSS--------WA--------GTVELYE-------- : 274
CiTA2b  : ASEANAVIISVDYRLAPEHLLPIAHEDSWAALQWIANHSEGQGPE--PWLNEHADFGRVFLAGESAGANIAQYVAVQAGAT-GL---AGPNIVGTLILHPYFGSKDSD--------------D---KMYKYMCPTSTGF-EDPLLYPEVD--PNLGRMAGGRVLVCVAEKDRLKDRGLGYCETLRQSS--------WA--------GTVELYE-------- : 274
VvTA    : VAAANLIAVSVDYRLAPEHPLPIAYDDSWAALQWISSHANGSGPE--PLFNHVDFGRVFLVGESAGANIAQHVAVRAGVT-GL---GGVKPVGLILAHPFFVG-KEP--------------D---KMIEFLYPSCSRVNDDPKLNPNVD--PNLSKMGCERVLVFVAEKDWLKSRGVGYCETLGKIG--------WT--------GAVELME-------- : 270
DKTA    : ASQARCIAVSVEYRLAPEHPLPIAHDDSFSALQWVAAHSTGQGPD--PWLNRYADFGRVFLAGESAGANLAHHVAVRTGAS-GL---GGVRLLGLVVAHPFFAG-KEP--------------D---KMMLYLYPGSSGSDDDPKLSPNAD--PDLGKMGCSRVLACVAEKDWLKPRGVAYCETLKKSG--------WK--------GTVELIE-------- : 271
CsTA1   : ASQAHLIAISVDYRLAPEHPLPIAYDDSWSALQWIATHSNGQGPD--PWLNQYADFGRVFLAGESAGANIAHQVAVRVGTV-GL---EGFMPRGVIIIHPYFAG-SEP--------------D---KMIQYLYPGSSGSEDDPNLSPKED--PNLTKMGCSKVIVFVAEKDRLKPRGVDYYETLKNSG--------WE--------GRVEFVE-------- : 267
CsTA2   : ASQAHLIAISVDYRLAPEHPLPIAYDDSWSALQWIATHSNGQGPD--PWLNQYADFGRVFLAGESAGANIAHQVAVRVGTV-GL---EGFMPRGVIIIHPYFAG-SEP--------------D---KMIQYLYPESSGSEDDPNLSPKED--PNLTKMGCSKVIVFVAEKDRLKLRGVDYYETLKNSG--------WE--------GRVEFVE-------- : 267
CsTA3   : ASQAHLIAISVEYRLAPEHPLPIAYEDSWSALQWIATHSNGQGPD--PWLNQYADFGRVFLAGESAGANIAHQVAVRVGTV-GL---EGFMPRGVIIIHPYFAG-SEP--------------D---KMIQYLYPGSSGSEDDPNLSPKED--PNLTKMGCSKVIVFVAEKDRLKPRGVDYYETLKNSG--------WE--------GRVEFVE-------- : 267
CsTA4   : ASQAHLIAISVDYRLAPEHPLPIAYEDSWSALQWIATHSNGQGPD--PWLNQYADFGRVFLAGESAGANIAHQVAVRVGTV-GL---EGFMPRGVIIIHPYFAG-SEP--------------D---KMIQYLYPGSSGSEDDPNLSPKED--PNLTKMGCSKVIVFVAEKDRLKPRGVDYYETLKNSG--------WE--------GRVEFVE-------- : 267
CsTA    : ASQAHLIAISVDYRLAPEHPLPIAYDDSWSALQWIATHSNGQGPD--PWLNQYADFGRVFLAGESAGANIAHQVAVRVGTV-GL---EGFMPRGVIIIHPYFAG-SEP--------------D---KMIQYLYPGSSGSEDDPNLSPKED--PNLTKMGCSKVIVFVAEKDRLKPRGVDYYETLKNSG--------WE--------GRVEFVE-------- : 267
CcTA-L1 : AAKANAIAVSVEYGLFPERPPPACYEDSWAGLQWIASHANGNGPE--PWLNEHADLNRVFVGGDSGGGNITHTLAVRVGSI-GL---PGLKVIGAILVHPYFGG-MED--------------D---EMWLYIFPTSGGL-EDPRLKPPAE---DLSRLGCERMLIFFAENDHLRGVGGSYYEELKKSG--------WG--------GSVEVVE-------- : 268
CiTA-L1 : VAKANVIAVSVEYGLFPTRPIPACYEDSWAALQWVASHSNRNGPE--PWLNDHADFQRVFIGGDSAGGNISHTQLVRVGSI-GL---PGVKVVGVVLVHPYFGG-TDD--------------D---RMWLHMCPTNGGL-EDRRLKPAAG---DLARLGCERVLIFVAEKDHLMDVGKRYYEELKKSG--------WG--------GSVEIVE-------- : 269
JrTA-L3 : VAKANVIAVSVEYGLFPTRPIPACYEDSWAALQWVASHSNRNGPE--PWLNDYADFQRVFIAGNSAGGNISHTLAVRVGSI-GL---PGVKVIGVVLLHPYFGG-TDD--------------D---RMWLHMCPTNGGL-EDPRLKPAAG---DLARLGCERVLIFVAEKDHLMDVGKRYYEELKKRSLRYNVGDMWSLEAVDACFGSLAFMKMECYISEI : 293
JrTA-L4 : VAKANVIAVSVEYGLFPTRPIPACYEDSWAALQWVASHSKRNGPE--PWLNDYADFQRVFIGGDSAGGNISHTLAVRVGSI-GL---PGVKVIGVVLLHPYFGG-TDD--------------D---RMWLHMCPTNGGL-EDPRLKPAAG---DLARLGCERVLIFVAEKDHLMDVGKHYYEELKKSG--------WG--------GSVEIVE-------- : 269
PtTA-L5 : AADANVIVVSVEYGLFPTRPIPACYEDSWEALQWVASHADGSGAE--PWLNNHADFGKVFLGGDSGGANISHTLAFRVGSI-GL---PGVKVVGMIMVHPFFGG-TED--------------D---KMWLYMCPSNSGL-DDPRLNPGLE---DLARLGCERVLIFVAEKDSLIAVGRNYYEKLKKSG--------WK--------GSVEIVE-------- : 270
PgTA-L1 : VAAADCVAVSVEYGLFPDRPLPACYEDSWAALQWVAAKQD-------PWIAEHVDLDRVFIAGNSAGGNISHTLACRVGSI-RL---SGVKVVGVILVHPYFGG-TGD--------------D---RMWLYMCPTNAGL-EDPRLKPSTA---DLARLGCERVLVFVAEKDHLMEVGIRYVEELKKSG--------WG--------GNVELVK-------- : 266
PgTA-L2 : AAQANCVAVSVEYGLFPDRPLPACYEDSWAALQWVATRQD-------PWIAEHADLNRVFLAGDSAGGNILYNLAARVGSV-GL---PGAKVVGAILVHPYFGG-TED--------------D---HMWLFMCPTNGGM-EDPRLKPAKE---DLARLGCERVLVFVAEKDHLMEVGIRYVEELKKSG--------WG--------GSVELVK-------- : 266
VvTA-L2 : VSEANIIAVSVEYGLFPDRPIPACYEDSWAALQWVASHASGDGPE--PWLNDYADFNRVFIAGDSAGGNISHTLAVRVGSI-GL---TGVRVVGVVLVHPYFGG-TDD--------------D---KMWLYMCPTNGGL-EDPRMKPAAE---DLARLGCEKVLVFVAEKDHLREVGWNYYEELKKSG--------WK--------GTVEIVE-------- : 268
VvTA-L3 : VSQGNAIAVSVEYGLFPDHPIPACYEDCAEALQWVASHAKGGGRE--PWLINHADFDRIFIVGDSAGGNISHTMAVRVGTI-GL---AGVRVVGVVMVHPFFGG-TID--------------D---EMWMYMCPTNGGL-EDPRMKPAAE---DLARLGCERMLLFVAEKDHLRDVGWRYYEELKKSE--------WI--------GTVEIVE-------- : 269
FvTA-L2 : SSSADVIAVSVEYGLFPTRPIPACYEDSWAALQWVATHADGTGSD--QWLNEYADFNRVFIGGDSAGGNITHTLVSRVGSV-GL---PGVKVVGAILMHPYFGG-TDD--------------D---KMWLYINKENEGL-QDRRLKPAAE---DLRRLGCEKVLVFVAEKDHLNGVGRSYVEGLKKSG--------WK--------GSVEMKE-------- : 272
FvTA-L3 : AASANVIAVSVEYGLFPTRPIPACYDDSWAALQWVAT----GGSE--PWLNDYADLTRVFVGGDSAGGNIAHTLLFRVGSI-GL---PGAKVVGAVLVHPYFGG-TDD--------------D---KMWLYMNKENEGL-QDRRLVPATE---DLRRLGCERVLVFVAEMDHLKGGGKRYMEELKKSG--------WT--------GSVEVEE-------- : 267
FvTA-L1 : AAEANAIAVSVEYGLFPDRPIPACYDDTWAALKWVQSHSTRSGPD--PWMNLYADLDRVFLCGDSGGANMCNNLAVRVGSV-GL---PGVKVAGMVMVHPFFGGLAVD--------------D---EMWLYMCPENGGV-QDRRLRPPAE---DLAKLGCERVLIFFAEKDHLREVGQWYCDELKKSE--------WG--------GSVEVVE-------- : 271
PtTA-L2 : VAEANVIAVSLEYGLWPERPLPGSYVDAWAGLKWIASHVKGNGPE--PWLNDNADFSRFFMGGDSGGANMSNFLAVQIGSY-GL---PGVRLIGMIMVHPFFGG-MED--------------D---EMWMFMYPTNCGK-QDPKLKPPPE---DLAKLGCEKVLVFLAEKDHLREVGGIFYEDLKRSG--------YK--------GALEVVE-------- : 270
JrTA-L1 : AAKANAIAVSVEYGLFPERPPPACYEDSWAGLQWIASHANGNGPE--PWLNEHADLNRVFVGGDSGGGNISHTLAVRVGSI-GL---PGLKVIGAILVHPYFGG-MED--------------D---EMWLYMFPTSGGL-EDPRLKPPVE---DLSRLGCERMLIFFAENDHLRGVGGSYYEELKNSG--------WG--------GSVEVVE-------- : 268
CcTA-L3 : VSEASVVAVSVDYRRAPEYPLPVAYDDSWAVLKWVESHADEKGPD--EWLKSHADFERVFLAGDSAGANIAHHMGLRVAAE-GL--HAGVKLEGIVLVHPYFWG-KEPVGSETAEAEKRAKID---DLWRFVYPATSGS-DDPYINPASD--PKLGSLGTGKVLICVAEKDLLKDRGWYYRVILGKSG--------WG--------GVVKVLE-------- : 286
CiTA-L3 : AAKANAIAVSVEYGLFPERPPPACYEDSWAGLQWIASHANGNGPE--PWLNEHADLNRVFVGGDSGGGNITHTLVVRVGSI-GL---PGLKVIGAILVHPFFGG-MED--------------D---EMWLYMFPTSGGL-EDPRLKPPAE---ELSRLGCERMLIFFAENDHLRGVGGSYYEELKKSG--------WG--------GSVEVVE-------- : 268
CiTA-L2 : AAESNLIAVSVEYRLAPEHPIPACYEDSWAGFQWVASHANGNGPE--SWLNDHADFNRVFLAGDSAGGNMSHNLAVRVGST-HI---PDVKLLGAILVHPYFGG-TDD--------------D---KMWLYMCPENSGL-DDPRLKPAAE---DLERLGCERLLVFVGGKDHLREVGWRYYKELKKSG--------WG--------GRVEIVE-------- : 331
CcTA-L2 : VSEASVVAVSVDYRRAPEHPLPVAYDDSWAVLKWVESHADEKGPH--EWLKSHADFERVFLAGDSAGANIAHHMGLRVGTE-GL--HAGVKLEGIVLVHPKFWG-KEPVGNETAWAEKRAKIE---DLWRFVYPATSGS-DDPYINPASD--PKLGSLGTGKVLICVAEKDLYKDRGWYYREILGKSG--------WG--------GVVKVFE-------- : 286
JrTA-L2 : AAESNLIAVSVEYRLAPEHPIPACYEDSWAGFQWVASHANGNGPE--SWLNDHADFNRVFLAGDSAGGNISHNLASRVGST-QI---PDVKLVGAILVHPYFGG-TDD--------------D---KMWLYMCPENSGL-DDPRLKPAAK---DLERLGCERVLVFVGGKDHLREVGWRYYKELKKSG--------WG--------GRVEIVE-------- : 218
CsTA-L1 : AAEAHVIAVSVEYRLAPEHPIPACYDDSWAALKWIGSHANGDGPD--LWLNNHADFNQVFVGGDSAGGNISHTMAFRAGTI-GL---PGIRLVGAFLVHPYFGGVELD--------------D---KMWLYMCPSNSGL-DDTRMKPSVE---DLGRLGCERVLVFVAEKDHLSGPGRAYYENLKISR--------WK--------GTVEIAE-------- : 272
CsTA-L2 : TGESTSIVVSVEYRLAPEHPIPACYDDSWAVLRWVAAHSTRDGAE--PWLNDHADFSRVFVGGDSAGGNMAHTLAYRVGTI-GL---PGVKLVGAFLTHPYFGGVEAD--------------D---KMWMYMCPTNSGF-DDPRMKPPVE---DLAVIGCERVLVFVAGNDHLREPGRAYYERLKRSG--------WK--------GSVEIVE-------- : 193
CsTA-L4 : AAESTSIVVSVEYRLAPEHPIPACYDDAWDVLRWVAAHSTRDGAE--PWLNDHADFSRVFVGGDSSGGNMAHTLAYRVGTI-GL---PGVKLVGAFMAHPYFGGVEED--------------D---KIWMYMCPTNSGF-DDPRMKPPVE---DLAVIGCERVLMFVAGNDPMREAGRAYYERLKKSG--------WK--------GSVEIVE-------- : 270
VvTA-L5 : VAEANVIALSVDYRLAPEHPIPACYDDSWAAVQWAASHANGDGPD--TWLNNHADFSRVFFAGDSAGGNISNTLAFRVGSS-GL---PGVKVVGVVLVHPYFGG-TGD--------------D---QMWLYMCPNHGGL-EDPRLKPGAE---DLARLGCERVLMFVAEKDHLRPVAWDYYEKLKKSE--------WK--------GTVEIVE-------- : 281
VvTA-L1 : VAEANVIGVSVEYRLAPENPIPACYDDSWAALQWVASHADGNGPE--PWLNSHSDMNRVFIAGDSAGGNIAHTLAVRVGSI-GL---PGAKVVGVVLVHPYFGG-TVD--------------D---EMWLYMCPTNSGL-EDPRLKPAAE---DLARLRCERVLIFVAEKDHLREIGWRYYEDLKKSG--------WK--------GTVEIVE-------- : 384
VvTA-L4 : VSQANVIGVSVEYRLAPEHPIPACYDDSWAALQWVASHANGNGPE--PWLNSYANLSRVFIAGDSAGGNISHTLMVRVGSL-GL---AGANVVGMVLVHPYFGG-TTD--------------D---GVWLYMCPNNGGL-EDPRLRPTAE---DMAMLGCGRVLVFLAENDHLRDVGWNYCEELKKSG--------WE--------GMVETVE-------- : 268
PtTA-L3 : VTEANVIAVSIEYRLAPEHPIPTGYEDSWTSLRWVASHANGHGPE--PWLNIHGDFSRVFLAGDSAGANIAYNLAVRIRSV--L---PKMKLAGMILMHPFFMG-SKL--------------D---KMWFYLCPRNGGL-KDSRFKPAAE---DMATTGCERVLVFLAGRDKLKNARGSYYEELKKCG--------WE--------GNVEMVE-------- : 272
FvTA-L4 : VAQANVIAVSVDYRLAPEHPIPACYEDSWAVLNWVASHANGDGPD--PWLNSHADFGRVFLAGDSAGANISHNIVSRVGST-GL---PGVKIVGVALVHPYFGG-TED--------------D---KMWLYMCPENGGL-DDPRLKPAVE---DLRRLGCERVLVFVAGKDHLRERGTSYVEALKGSG--------WK--------GTVEIVE-------- : 274
JrTA-L6 : SAKASAIAVSVEYRLAPEHPIPACYEDSWAALQWVASHANGDGPD--PWLNDHADFRRVFLAGDSAGANISHQLVVRLGSC-GL---PGVKLSGMALVHPYFGG-TED--------------D---KMWLYMFPENRGP-EDPRLKPAAE---DLTGLGCERVLIFVAAEDHLRERGTGYHEDLKKSG--------WK--------GTVEIVE-------- : 272
CsTA-L3 : AAAANVLAVSIEYRLAPEHPVPACYDDSWSVLQWVTRHANGSIPDPDPWLVDHADFTRVFLAGDSAGANIAHNMAVRAGVD-GL--GSGLKIVGVVLVQPFFGN-DEI--------------D---KLWMLICPETSGI-NDPRLNPAAD-PGLDAKLGCTRVLVCVAGKDHLRERGWSYYEALKKSE--------WG--------GSVEIEE-------- : 274
VvTA-L6 : ASAANVIAVSVHYRRPPEHPIPIPHDDTWDAFQWVAAHSSGQGPE--PWLNHHAKFDRVFFAGDSAGANIAHNMAIRAGTT-QP---PNVKIYGIVLVHPYFGN-NGP--------------D---RLWNYLCP--SGV-HNLLFDPAVD--TKLSILGCGKVLIFVAGKDVLKDRGCFYYEAVKKSG--------WG--------GAVEMVE-------- : 269
FvTA-L5 : AAQANVVALSIHYRRAPEHPLPAAFEDSWAAFQWAATHSTGGGPE--SWLNEHADFNRVFVAGDSAGSTLAHHVVRQSGVE-GV---SGVNTVGVVLFHPYFRN-EVP--------------D---KLLEVIYPTGGGL-EDPKMYPGND--PRLGSLGAGRVLVFVAEKDFLKDRGWSYYEALKKSG--------WS--------GVVEIEE-------- : 272
JrTA-L5 : SAEANVVAVSIHYRRAPEHPLPIAYDDTWAAVQWMASHSKGKGPE--TWLNDHVDFERVFLAGDSAGANLAHSTARRAAVD-GL---GEVKIVGMVLIHPYFGN-DQR--------------S---KMLEIIFPTMSGS-DDPRMYPNKD--SKLSRLGCSRVLVLVAENDGLKERGWTYYEALRKSG--------WS--------GAVDIME-------- : 270
PtTA-L1 : AHQANVIAVSVQYRRAPEHPLPIAYDDSWAAIQWVASHVNGIGVE--SWLNKHADFERTFLAGDSAGANIAHNMTVRAGVN-GL---FGVKTVGMVLAHPFFGG-KEP--------------DFFSPVIEYIFPDVKIY-DDPRINPAGAGGVELASLGCSRVLIFVAGNDGLRERGYSYYDALKKSG--------WS--------GVVEIVE-------- : 277
PtTA-L4 : VHNANVIAVSVQYRRAPEHPLPAAYDDSWAAIQWVASHVNGEGSE--SWLNGHADFDRTFLAGDSAGANIAHNMAVRAGTTNGL---NGVKIVGVVLAHPFFGN-NEP--------------DTFSPVIEFIFPSVRIY-DDPRINPAGAGGAELASLGCDLRDFSRVVRVDLMRTS-------------------------------------------- : 257
        ............a.....sv.Y.l.Pe.p.P..y.Dsw..l.W.a.h....gp....wln..ad..rvf..G.SaG.ni.h..a...g...gl....g....g....hP.F..................d.........p...g...dp...P.......l...gc..vl..vae.D.l...g..y.e.l..s.........w.........g.ve..e.............
```

```
          ................*.......460.........*.......480.........*.......500.........*.......520.........*.......540.........*.......560.........*.......580.........*.......600.........*.......620.........*.......640.........*.......660.........
JrTA1   : -------SKGEDHCFHLF------------------------------------------------NAESEEAAQLMRKFGD------------------------------------------------------------------------------------------------------------------------------------------ : 298
CcTA1   : -------SKGEDHCFHLF------------------------------------------------NAESEEAAQLMKKFVD------------------------------------------------------------------------------------------------------------------------------------------ : 298
CiTA1   : -------SKGEDHCFHLF------------------------------------------------NAESEEAAQLMKKFVD------------------------------------------------------------------------------------------------------------------------------------------ : 363
FvTA    : -------TKGEDHCFHMF----------------------------------------------NPNPNIDKAGDLLLKIVD------------------------------------------------------------------------------------------------------------------------------------------ : 301
FaTA    : -------TKGEDHCFHMF----------------------------------------------NPNPNIDKAGDLLQKIVD------------------------------------------------------------------------------------------------------------------------------------------ : 301
PgTA1   : -------NKEEDHCFHLF------------------------------------------------KRN-ENTKLLMKKLAE------------------------------------------------------------------------------------------------------------------------------------------ : 305
PgTA2   : -------AKGEDHCFHMF------------------------------------------------KRN-ERAEQLLQRMAD------------------------------------------------------------------------------------------------------------------------------------------ : 353
PtTA2   : -------TKGEDHCFNAF------------------------------------------------K-QCGETDALNKKVVD------------------------------------------------------------------------------------------------------------------------------------------ : 296
CclTA   : -------TSGEDHCFHMF------------------------------------------------RPDSEKVGPLIEKLVH------------------------------------------------------------------------------------------------------------------------------------------ : 298
PtTA1   : -------TKGEDHCFHFF------------------------------------------------NPKSENIGPLMKKMVD------------------------------------------------------------------------------------------------------------------------------------------ : 298
JrTA2   : -------TEGEGHGFFLL------------------------------------------------NPTSDKVEPLVEVLVD------------------------------------------------------------------------------------------------------------------------------------------ : 301
CcTA2   : -------TEGEGHGFFLL------------------------------------------------NPTSDKVEPLVKVLVD------------------------------------------------------------------------------------------------------------------------------------------ : 301
CiTA2a  : -------TEGEGHGFFLL------------------------------------------------NPTSDKVEPLVKVLVD------------------------------------------------------------------------------------------------------------------------------------------ : 301
CiTA2b  : -------TEGEGHGFFLL------------------------------------------------NPTSDKVEPLVKVLVD------------------------------------------------------------------------------------------------------------------------------------------ : 301
VvTA    : -------NEGEDHCFHLF------------------------------------------------NSDSEKAEMLMKRTVS------------------------------------------------------------------------------------------------------------------------------------------ : 297
DKTA    : -------SEGEDHCFHVF------------------------------------------------NPNCEKAEALMQKLVS------------------------------------------------------------------------------------------------------------------------------------------ : 298
CsTA1   : -------DKGEDHCFHMF------------------------------------------------SPNSDKVVGLIQKLGT------------------------------------------------------------------------------------------------------------------------------------------ : 294
CsTA2   : -------DKGEDHCFHMF------------------------------------------------SPNSDKVVGLMQKLGT------------------------------------------------------------------------------------------------------------------------------------------ : 294
CsTA3   : -------DKGEDHCFHMF------------------------------------------------SPNSDKVVGLIQKLGT------------------------------------------------------------------------------------------------------------------------------------------ : 294
CsTA4   : -------DKGEDHCFHMF------------------------------------------------SPNSDKVVGLMQKLGT------------------------------------------------------------------------------------------------------------------------------------------ : 294
CsTA    : -------DKGEDHCFHMF------------------------------------------------SPNSDKVVGLMQKLGT------------------------------------------------------------------------------------------------------------------------------------------ : 294
CcTA-L1 : -------HQGEDHVFHLT------------------------------------------------KPDSENAVDLVNKFAS------------------------------------------------------------------------------------------------------------------------------------------ : 295
CiTA-L1 : -------NLGEEHCFHLF------------------------------------------------NLKYDKAVAVIDKFVS------------------------------------------------------------------------------------------------------------------------------------------ : 296
JrTA-L3 : YRGSPLLQLTQLICLYCLMALGRMEATDLLSCRAQSFSDLLALQTAKNHREPPAPPEQSKTAPTVHPSLQVERGLRKQHRF------------------------------------------------------------------------------------------------------------------------------------------- : 374
JrTA-L4 : -------NLGEEHCFHLF------------------------------------------------NLRYDKAVAVIDKFVS------------------------------------------------------------------------------------------------------------------------------------------ : 296
PtTA-L5 : -------NEDEDHCFYLL------------------------------------------------DLNSEKAVELLHKFVS------------------------------------------------------------------------------------------------------------------------------------------ : 297
PgTA-L1 : -------NVGEPHSFHMS------------------------------------------------DPNYEKAVELKNKFAS------------------------------------------------------------------------------------------------------------------------------------------ : 293
PgTA-L2 : -------NLGEEHCFHLF------------------------------------------------DPKYEKAVELKNKFVS------------------------------------------------------------------------------------------------------------------------------------------ : 293
VvTA-L2 : -------NHGEEHCFHLH------------------------------------------------DLSYEKSVDLIKQIAS------------------------------------------------------------------------------------------------------------------------------------------ : 295
VvTA-L3 : -------NHGEEHCFHRR------------------------------------------------DLTYEKAVALIHRIVS------------------------------------------------------------------------------------------------------------------------------------------ : 296
FvTA-L2 : -------NKGKEHCFHLH------------------------------------------------ETKDEQALDLIKTLSSNAISPPSSLPLSPPHHRTTSPLNPTRPFLQICLQSRSLTTQPILNDAVTHEFRFFRVYESGRVEKFHQTHKIPPKDDPVTGVRSKDVVISSEPAISARVFLPQIRDPTRKLPVLFHVHGGGFSFESAFSPHIHNYVST : 437
FvTA-L3 : -------TVGKDHCFHLA------------------------------------------------EPNDELTTDLIKKNG------------------------------------------------------------------------------------------------------------------------------------------- : 293
FvTA-L1 : -------HEDEFHEFHLL-----------------------------------------------SEPEGDNALDLIKRFGS------------------------------------------------------------------------------------------------------------------------------------------ : 299
PtTA-L2 : -------HEGVAHEFHLF------------------------------------------------DPAHDKSLSLVKKFAS------------------------------------------------------------------------------------------------------------------------------------------ : 297
JrTA-L1 : -------HRGEDHVFHLM------------------------------------------------KPDSENAVDLLNKFAS------------------------------------------------------------------------------------------------------------------------------------------ : 295
CcTA-L3 : -------SAGEDHVFHLL------------------------------------------------NPACENAKALLQRIVS------------------------------------------------------------------------------------------------------------------------------------------ : 313
CiTA-L3 : -------HQGEDHIFHLM------------------------------------------------KPDSENAIDLVNKFAS------------------------------------------------------------------------------------------------------------------------------------------ : 295
CiTA-L2 : -------NEAEDHCFHLD------------------------------------------------DIKQEKAVALIKRFAS------------------------------------------------------------------------------------------------------------------------------------------ : 358
CcTA-L2 : -------SAGEGHVFHLK------------------------------------------------NPACENAKALLQRIVS------------------------------------------------------------------------------------------------------------------------------------------ : 313
JrTA-L2 : -------NEGEDHCFHLD------------------------------------------------DLKQEKAVALIKRFAS------------------------------------------------------------------------------------------------------------------------------------------ : 245
CsTA-L1 : -------NDGEEHCFHLH------------------------------------------------HTTSDNAVALIKKIVS------------------------------------------------------------------------------------------------------------------------------------------ : 299
CsTA-L2 : -------NEGQEHCFHLH------------------------------------------------DPTNENAVALVKKIVD------------------------------------------------------------------------------------------------------------------------------------------ : 220
CsTA-L4 : -------NVGQDHCFHLQ------------------------------------------------DPTNENAVALVKKIVD------------------------------------------------------------------------------------------------------------------------------------------ : 297
VvTA-L5 : -------NHGEEHVFHLM------------------------------------------------NPKCENAAVLMKKIVS------------------------------------------------------------------------------------------------------------------------------------------ : 308
VvTA-L1 : -------NHGEEHGFHLD------------------------------------------------NLTGDQTVDLIARFES------------------------------------------------------------------------------------------------------------------------------------------ : 411
VvTA-L4 : -------NHGERHVFHLM------------------------------------------------NPRCENAATLMGKIVS------------------------------------------------------------------------------------------------------------------------------------------ : 295
PtTA-L3 : -------HDGVGHVFHLL------------------------------------------------KPRCEQALDMMKRLVS------------------------------------------------------------------------------------------------------------------------------------------ : 299
FvTA-L4 : -------HEEENHVFHLM------------------------------------------------KPESENAVDLMSRLVS------------------------------------------------------------------------------------------------------------------------------------------ : 301
JrTA-L6 : -------HEEEDHVFHLR------------------------------------------------KPNCEKALDLVDRLAS------------------------------------------------------------------------------------------------------------------------------------------ : 299
CsTA-L3 : -------TEGEPHVFHLF------------------------------------------------DPACEKARTLMKRVVS------------------------------------------------------------------------------------------------------------------------------------------ : 301
VvTA-L6 : -------SEGEEHVFHLF------------------------------------------------NPDCDKARALIQKFAS------------------------------------------------------------------------------------------------------------------------------------------ : 296
FvTA-L5 : -------TEGEDHVFHLF------------------------------------------------DPSCEKAVSLVKKVAA------------------------------------------------------------------------------------------------------------------------------------------ : 299
JrTA-L5 : -------TKGEDHVFHLF------------------------------------------------NPNSENASSLLKQMAS------------------------------------------------------------------------------------------------------------------------------------------ : 297
PtTA-L1 : -------TEGEDHVFHLF------------------------------------------------NPDCDKAVFMMKLVVS------------------------------------------------------------------------------------------------------------------------------------------ : 304
PtTA-L4 : ---------------------------------------------------------------------------------------------------------------------------------------------------------------------------------------------------------------------------- :  -
          ..................ge.h.fh..........................................................l.....................................................................................................................................
```

```
                 ................*.......680.........*.......700.........*.......720.........*.......740.........*.......760.........*.......780.........*.......800.........*...........
JrTA1    : ----------------------------------------------------------FVAGT----------------------------------------------------------------------------------------- : 303
CcTA1    : ----------------------------------------------------------FVAGE----------------------------------------------------------------------------------------- : 303
CiTA1    : ----------------------------------------------------------FVSGE----------------------------------------------------------------------------------------- : 368
FvTA     : ----------------------------------------------------------FVTQC----------------------------------------------------------------------------------------- : 306
FaTA     : ----------------------------------------------------------FVTQC----------------------------------------------------------------------------------------- : 306
PgTA1    : ----------------------------------------------------------FVNLP----------------------------------------------------------------------------------------- : 310
PgTA2    : ----------------------------------------------------------FINRE----------------------------------------------------------------------------------------- : 358
PtTA2    : ----------------------------------------------------------FMTME----------------------------------------------------------------------------------------- : 301
CclTA    : ----------------------------------------------------------FINNAWT--------------------------------------------------------------------------------------- : 305
PtTA1    : ----------------------------------------------------------FIQLN----------------------------------------------------------------------------------------- : 303
JrTA2    : ----------------------------------------------------------FIKHR----------------------------------------------------------------------------------------- : 306
CcTA2    : ----------------------------------------------------------FIKHR----------------------------------------------------------------------------------------- : 306
CiTA2a   : ----------------------------------------------------------FIKHH----------------------------------------------------------------------------------------- : 306
CiTA2b   : ----------------------------------------------------------FIKHH----------------------------------------------------------------------------------------- : 306
VvTA     : ----------------------------------------------------------FINQE----------------------------------------------------------------------------------------- : 302
DKTA     : ----------------------------------------------------------FVNQD----------------------------------------------------------------------------------------- : 303
CsTA1    : ----------------------------------------------------------FVNEN----------------------------------------------------------------------------------------- : 299
CsTA2    : ----------------------------------------------------------FVNEN----------------------------------------------------------------------------------------- : 299
CsTA3    : ----------------------------------------------------------FVNEN----------------------------------------------------------------------------------------- : 299
CsTA4    : ----------------------------------------------------------FVNEN----------------------------------------------------------------------------------------- : 299
CsTA     : ----------------------------------------------------------FVNEN----------------------------------------------------------------------------------------- : 299
CcTA-L1  : ----------------------------------------------------------FIVQG----------------------------------------------------------------------------------------- : 300
CiTA-L1  : ----------------------------------------------------------FVKQDH---------------------------------------------------------------------------------------- : 302
JrTA-L3  : ----------------------------------------------------------FSVTDRLPCCTPSPPCLAAGCPIVSHRRARLLHSVPISASDNFWKQQ----------------------------------------------- : 421
JrTA-L4  : ----------------------------------------------------------FVIQDR---------------------------------------------------------------------------------------- : 302
PtTA-L5  : ----------------------------------------------------------FLKQD----------------------------------------------------------------------------------------- : 302
PgTA-L1  : ----------------------------------------------------------FIRP------------------------------------------------------------------------------------------ : 297
PgTA-L2  : ----------------------------------------------------------FITQ------------------------------------------------------------------------------------------ : 297
VvTA-L2  : ----------------------------------------------------------FINRE----------------------------------------------------------------------------------------- : 300
VvTA-L3  : ----------------------------------------------------------FIKQS----------------------------------------------------------------------------------------- : 301
FvTA-L2  : LSSEADVIAVSVEYRLAPEHPIPACYDDPWAALNWVSSHCNRNGPEPWLNEHADFERVFVAGDSAGGNISHDLAVRVGSEDDAMWLYMCRDNNGLQDCRLRPSVEDLRRLGCERVLVFVAEKDHLNGVGKNYVEELRKSGWEGSVEIVENEG : 589
FvTA-L3  : ----------------------------------------------------------FVY------------------------------------------------------------------------------------------- : 296
FvTA-L1  : ----------------------------------------------------------FINQN----------------------------------------------------------------------------------------- : 304
PtTA-L2  : ----------------------------------------------------------FLNEV----------------------------------------------------------------------------------------- : 302
JrTA-L1  : ----------------------------------------------------------FIVQV----------------------------------------------------------------------------------------- : 300
CcTA-L3  : ----------------------------------------------------------LINQGEL--------------------------------------------------------------------------------------- : 320
CiTA-L3  : ----------------------------------------------------------FIVHG----------------------------------------------------------------------------------------- : 300
CiTA-L2  : ----------------------------------------------------------FINQE----------------------------------------------------------------------------------------- : 363
CcTA-L2  : ----------------------------------------------------------LIKQGEL--------------------------------------------------------------------------------------- : 320
JrTA-L2  : ----------------------------------------------------------FINQEGAGAPI----------------------------------------------------------------------------------- : 256
CsTA-L1  : ----------------------------------------------------------FINQE----------------------------------------------------------------------------------------- : 304
CsTA-L2  : ----------------------------------------------------------FINQ------------------------------------------------------------------------------------------ : 224
CsTA-L4  : ----------------------------------------------------------FINQK----------------------------------------------------------------------------------------- : 302
VvTA-L5  : ----------------------------------------------------------FLNQE----------------------------------------------------------------------------------------- : 313
VvTA-L1  : ----------------------------------------------------------FINKD----------------------------------------------------------------------------------------- : 416
VvTA-L4  : ----------------------------------------------------------FLNQE----------------------------------------------------------------------------------------- : 300
PtTA-L3  : ----------------------------------------------------------FINQEPVIISWTSKI------------------------------------------------------------------------------- : 314
FvTA-L4  : ----------------------------------------------------------FINQAG---------------------------------------------------------------------------------------- : 307
JrTA-L6  : ----------------------------------------------------------FVKQG----------------------------------------------------------------------------------------- : 304
CsTA-L3  : ----------------------------------------------------------FMNQD----------------------------------------------------------------------------------------- : 306
VvTA-L6  : ----------------------------------------------------------FMNQD----------------------------------------------------------------------------------------- : 301
FvTA-L5  : ----------------------------------------------------------FMNQE----------------------------------------------------------------------------------------- : 304
JrTA-L5  : ----------------------------------------------------------FFNQN----------------------------------------------------------------------------------------- : 302
PtTA-L1  : ----------------------------------------------------------FINPVP---------------------------------------------------------------------------------------- : 310
PtTA-L4  : -------------------------------------------------------------------------------------------------------------------------------------------------------- :   -
           ..........................................................f.............................................................................................
```
