# Supplementary figures and images for: Genome-Wide Identification of Tannase Genes and Their Function of Wound Response and Astringent Substances Accumulation in Juglandaceae
Source: Front Plant Sci. 2021 May 17;12:664470. doi: 10.3389/fpls.2021.664470 (PMC8165273; doi:10.3389/fpls.2021.664470)

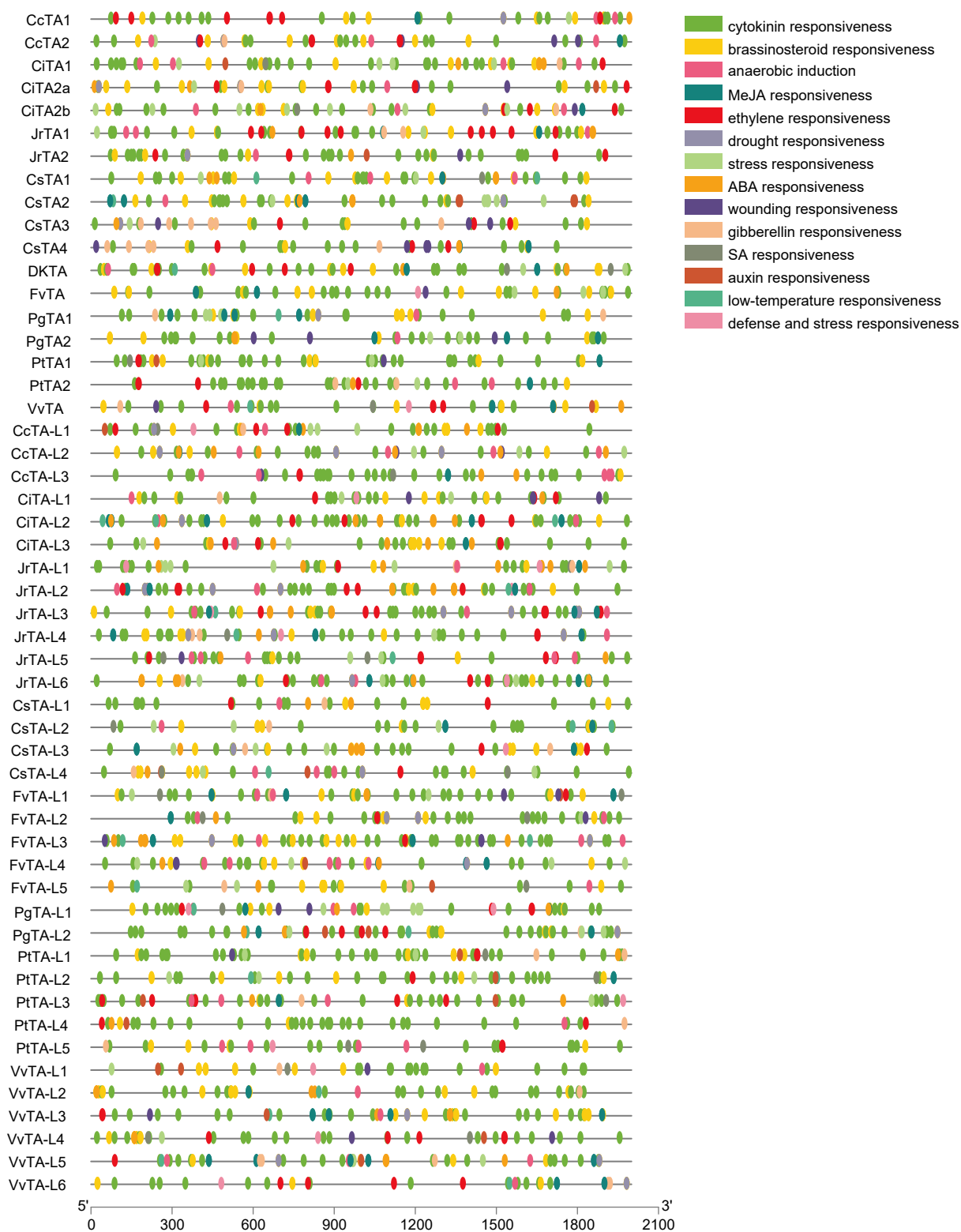

Supplement: Supplementary Figure 2 — Prediction of cis-acting elements in TA gene promoter regions. All cis-acting elements were marked with different colors according to the potential biological process. [file Data_Sheet_2.PDF]
