# Supplementary material for: Genome-Wide Identification of Tannase Genes and Their Function of Wound Response and Astringent Substances Accumulation in Juglandaceae
Source: Front Plant Sci. 2021 May 17;12:664470. doi: 10.3389/fpls.2021.664470 (PMC8165273; doi:10.3389/fpls.2021.664470)
Supplement: Supplementary Figure 3 — Scaffold locations and tandem repeat of TA genes in plants. The TA and TA-like genes were marked with red. Scaffold numbers are shown on the left side of each respective scaffold. The different genes are located according to their genomic position. [file Data_Sheet_3.PDF]

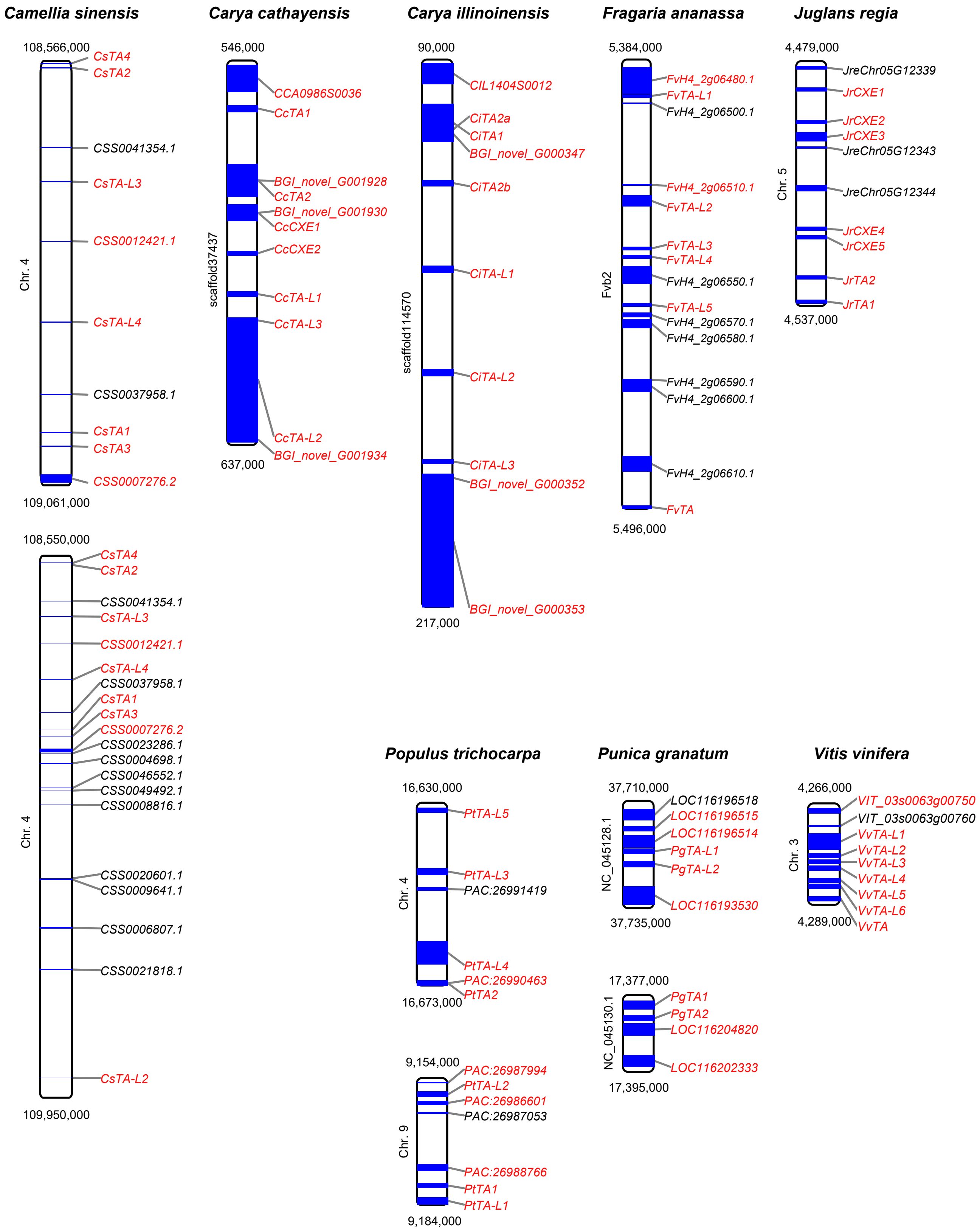

Camellia sinensis
108,566,000
CsTA4
CsTA2
CSS0041354.1
CsTA-L3
CSS0012421.1
Chr. 4
CsTA-L4
CSS0037958.1
CsTA1
CsTA3
CSS0007276.2
109,061,000
108,550,000
CsTA4
CsTA2
CSS0041354.1
CsTA-L3
CSS0012421.1
CsTA-L4
CSS0037958.1
CsTA1
CsTA3
CSS0007276.2
CSS0023286.1
CSS0004698.1
CSS0046552.1
CSS0049492.1
CSS0008816.1
Chr. 4
CSS0020601.1
CSS0009641.1
CSS0006807.1
CSS0021818.1
CsTA-L2
109,950,000
Carya cathayensis
546,000
CCA0986S0036
CcTA1
BGI_novel_G001928
CcTA2
BGI_novel_G001930
CcCXE1
scaffold37437
CcCXE2
CcTA-L1
CcTA-L3
CcTA-L2
BGI_novel_G001934
637,000
Carya illinoinensis
90,000
CIL1404S0012
CiTA2a
CiTA1
BGI_novel_G000347
CiTA2b
CiTA-L1
scaffold114570
CiTA-L2
CiTA-L3
BGI_novel_G000352
BGI_novel_G000353
217,000
Fragaria ananassa
5,384,000
FvH4_2g06480.1
FvTA-L1
FvH4_2g06500.1
FvH4_2g06510.1
FvTA-L2
FvTA-L3
FvTA-L4
Fvb2
FvH4_2g06550.1
FvTA-L5
FvH4_2g06570.1
FvH4_2g06580.1
FvH4_2g06590.1
FvH4_2g06600.1
FvH4_2g06610.1
FvTA
5,496,000
Juglans regia
4,479,000
JreChr05G12339
JrCXE1
JrCXE2
JrCXE3
JreChr05G12343
Chr. 5
JreChr05G12344
JrCXE4
JrCXE5
JrTA2
JrTA1
4,537,000
Populus trichocarpa
16,630,000
PtTA-L5
Chr. 4
PtTA-L3
PAC:26991419
PtTA-L4
PAC:26990463
PtTA2
16,673,000
9,154,000
PAC:26987994
PtTA-L2
PAC:26986601
PAC:26987053
Chr. 9
PAC:26988766
PtTA1
PtTA-L1
9,184,000
Punica granatum
37,710,000
LOC116196518
LOC116196515
LOC116196514
NC_045128.1
PgTA-L1
PgTA-L2
LOC116193530
37,735,000
17,377,000
PgTA1
PgTA2
NC_045130.1
LOC116204820
LOC116202333
17,395,000
Vitis vinifera
4,266,000
VIT_03s0063g00750
VIT_03s0063g00760
VvTA-L1
Chr. 3
VvTA-L2
VvTA-L3
VvTA-L4
VvTA-L5
VvTA-L6
4,289,000
VvTA
